# Flaxseed Reduces Cancer Risk by Altering Bioenergetic Pathways in Liver: Connecting SAM Biosynthesis to Cellular Energy

**DOI:** 10.3390/metabo13080945

**Published:** 2023-08-14

**Authors:** William C. Weston, Karen H. Hales, Dale B. Hales

**Affiliations:** 1 Department of Molecular, Cellular & Systemic Physiology, School of Medicine, Southern Illinois University, Carbondale, IL 62901, USA; william.c.weston1@gmail.com; 2 Department of Obstetrics & Gynecology, School of Medicine, Southern Illinois University, Carbondale, IL 62901, USA; khales@siumed.edu

**Keywords:** flaxseed, metformin, cancer, chicken, diabetes, NAFLD, obesity, one-carbon metabolism, bioenergetics, SAM

## Abstract

This article illustrates how dietary flaxseed can be used to reduce cancer risk, specifically by attenuating obesity, type 2 diabetes, and non-alcoholic fatty liver disease (NAFLD). We utilize a targeted metabolomics dataset in combination with a reanalysis of past work to investigate the “metabo-bioenergetic” adaptations that occur in White Leghorn laying hens while consuming dietary flaxseed. Recently, we revealed how the anti-vitamin B6 effects of flaxseed augment one-carbon metabolism in a manner that accelerates S-adenosylmethionine (SAM) biosynthesis. Researchers recently showed that accelerated SAM biosynthesis activates the cell’s master energy sensor, AMP-activated protein kinase (AMPK). Our paper provides evidence that flaxseed upregulates mitochondrial fatty acid oxidation and glycolysis in liver, concomitant with the attenuation of lipogenesis and polyamine biosynthesis. Defatted flaxseed likely functions as a metformin homologue by upregulating hepatic glucose uptake and pyruvate flux through the pyruvate dehydrogenase complex (PDC) in laying hens. In contrast, whole flaxseed appears to attenuate liver steatosis and body mass by modifying mitochondrial fatty acid oxidation and lipogenesis. Several acylcarnitine moieties indicate Randle cycle adaptations that protect mitochondria from metabolic overload when hens consume flaxseed. We also discuss a paradoxical finding whereby flaxseed induces the highest glycated hemoglobin percentage (HbA1c%) ever recorded in birds, and we suspect that hyperglycemia is not the cause. In conclusion, flaxseed modifies bioenergetic pathways to attenuate the risk of obesity, type 2 diabetes, and NAFLD, possibly downstream of SAM biosynthesis. These findings, if reproducible in humans, can be used to lower cancer risk within the general population.

## 1. Introduction

### 1.1. Obesity, Type 2 Diabetes, and Non-Alcoholic Fatty Liver Disease as Cancer Risk Factors

Since the 1970s, the obesity rate in the United States has more than doubled, and there is no socioeconomic class that protects individuals from obesity [1]. Approximately 33.7% of males and 38% of females in the United States can be classified as obese [2]. After considering genetics, nuclear radiation, and chronological age, the single greatest risk factor for cancer is obesity [3,4,5,6]. Obesity enhances cancer risk, in part, by causing chronic, low-grade inflammation that recruits tumor-associated macrophages to the tumor microenvironment [7,8,9,10,11,12,13,14]. Making matters worse, obesity is a primary risk factor for the development of type 2 diabetes, and most forms of cancer have a greater likelihood of occurrence in type 2 diabetics [15,16,17,18,19]. Obesity and diabetes also increase the risk of developing non-alcoholic fatty liver disease (NAFLD) [20,21]. NAFLD, in turn, exacerbates pre-existing diabetic complications [22,23]. The public needs a simple solution that can mitigate each of these intertwined pathologies.

### 1.2. Flaxseed’s Role as an Anti-Cancer Food

Human trials indicate that dietary flaxseed (30 g daily) helps to attenuate BMI [24,25], blood glucose [26], blood insulin [26], glycated hemoglobin (HbA1c%) [27], metabolic syndrome [28,29,30], and NAFLD [30,31,32,33]. These observations illustrate flaxseed’s potential to protect humans from cancer-prone states. However, researchers have not fully clarified the bioenergetic alterations that occur in response to flaxseed consumption. By gaining a better understanding of flaxseed’s effect on animal metabolism, we can help researchers to appreciate flaxseed’s potential as an anti-cancer food.

Our lab specializes in the investigation of flaxseed as an ovarian cancer intervention for White Leghorn laying hens. Laying hens provide a unique model for ovarian cancer research because laying hens develop biologically natural ovarian tumors beginning at two years of age [34,35,36]. Additionally, ovarian cancer’s mutational landscape, histopathology, and causal etiology are similar between humans and laying hens [34,37,38]. Our lab’s findings indicate that dietary flaxseed protects laying hens from ovarian cancer by attenuating systemic prostaglandin E2 [36], tumor estrogen receptor expression [39], tumor microRNA-200 expression [40], and tumor survival [41]. We recently observed a novel anti-cancer mechanism whereby flaxseed augments one-carbon metabolism in the laying hen [42]. Our present work extends that one-carbon metabolic framework by investigating the bioenergetic pathways that are modified when hens consume flaxseed.

### 1.3. Vitamin B6, 1-Amino D-Proline, and Flaxseed’s Effect on One-Carbon Metabolism

The term “vitamin B6” refers to six different vitamin B6 isoforms (sometimes called “vitamers”) that are based on three structures: pyridoxine, pyridoxal, and pyridoxamine. The pyridoxal moiety, having a reactive aldehyde at the 4′ carbon, can form a Schiff base with vitamin B6-dependent enzymes [43]. However, pyridoxal 5′-phosphate (PLP) is the cofactor that causes enzyme activation [44]. Over 140 PLP-dependent enzymatic reactions are known in biology, comprising 4% of all enzyme reactions [45,46]. Related specifically to our work, PLP is the obligate cofactor for the transsulfuration pathway. In animals, transsulfuration is regulated by two PLP-dependent enzymes: cystathionine beta synthase (CBS) and cystathionase (CSE). CBS oxidizes homocysteine to cystathionine and CSE oxidizes cystathionine to cysteine.

**Figure 1:**
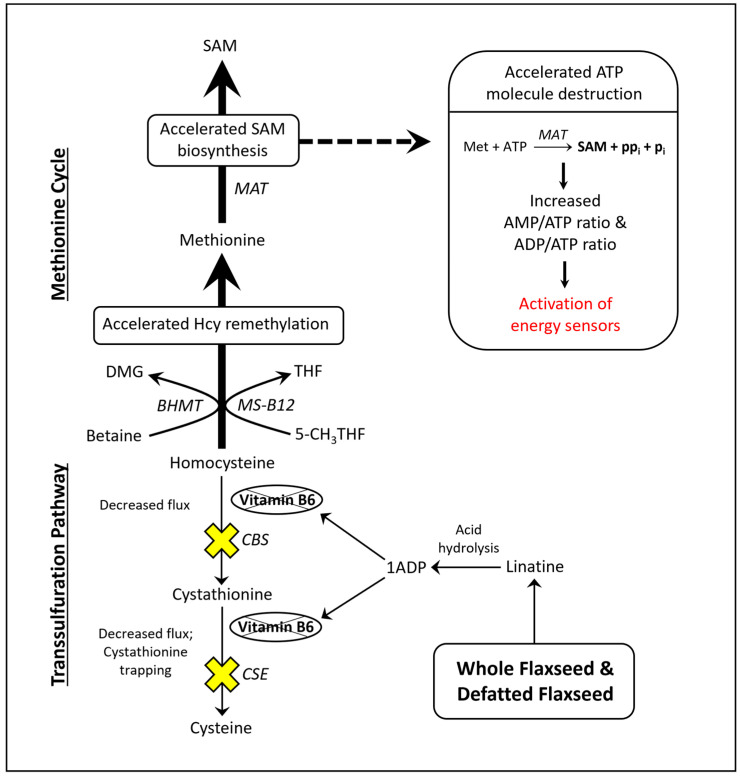
Model illustrating how flaxseed augments one-carbon metabolism in a manner that accelerates SAM biosynthesis, resulting in an elevated AMP/ATP ratio and an elevated ADP/ATP ratio (adapted from [42]). 1ADP = 1-amino D-proline, 5-CH_3_THF = 5-methyl tetrahydrofolate, BHMT = betaine homocysteine methyltransferase, CBS = cystathionine beta synthase, CSE = cystathionase, DMG = dimethylglycine, Hcy = homocysteine, MAT = methionine adenosyltransferase, Met = methionine, MS-B12 = methionine synthase complexed with vitamin B12, SAM = S-adenosylmethionine, THF = tetrahydrofolate.

Flaxseed contains a vitamin B6 antagonist “precursory” molecule known as linatine, which in the presence of hydrochloric acid undergoes hydrolysis to yield glutamic acid and 1-amino D-proline (1ADP) [47]. The amino group of 1ADP traps the 4′ carbonyl of pyridoxal or PLP, resulting in the formation of a covalently stable conjugate that cannot support PLP-dependent reactions [47]. Therefore, in nature, 1ADP insults vitamin B6 metabolism by attenuating the bioavailability of vitamin B6 [42,48,49]. In laying hens, we recently showed that flaxseed attenuates plasma 4-pyridoxic acid and causes an extreme perturbation of the transsulfuration pathway (i.e., 15-fold elevated cystathionine) [42]. Interestingly, homocysteine was not elevated despite such an extreme increase in cystathionine. To account for this, we observed evidence that flaxseed accelerates flux through the homocysteine remethylation pathway, culminating in the accelerated biosynthesis of S-adenosylmethionine (SAM) [42]. We illustrate these effects in Figure 1.

### 1.4. SAM Biosynthesis and AMPK Activation

Researchers illustrated that the acceleration of SAM biosynthesis can independently activate AMP-activated protein kinase (AMPK) [50]. SAM biosynthesis is regulated by the ATP-consuming enzyme methionine adenosyltransferase (MAT). MAT is unique because it fully consumes ATP without generating ADP, yielding SAM, pyrophosphate, and inorganic phosphate (Figure 1). By fully consuming ATP, MAT can readily elevate the AMP/ATP ratio and ADP/ATP ratio. Those ratios determine the rate at which AMP and ADP bind to the regulatory subunits of energy sensing proteins like AMPK. AMPK is activated, in part, by the increased binding of AMP and ADP to its γ-regulatory subunits [51,52]. During states of ATP deficiency, AMPK increases ATP synthesis by accelerating mitochondrial fatty acid oxidation (FAO) or glycolysis [53,54,55]. AMPK can also act to conserve ATP by inhibiting anabolic enzymes like acetyl-CoA carboxylase (ACC) and ornithine decarboxylase (ODC) [56,57,58]. In Supplementary Figure S1, we propose a novel means by which SAM biosynthesis should enhance the binding of AMP onto energy sensing proteins.

Downstream of glycolysis, the pyruvate dehydrogenase complex (PDC) serves as the primary checkpoint for pyruvate’s entry into the mitochondria. PDC oxidatively decarboxylates pyruvate to form acetyl-CoA, and acetyl-CoA then undergoes a condensation reaction with oxaloacetate to form citrate in the tricarboxylic acid (TCA) cycle [59]. Together, PDC and mitochondrial FAO contribute the vast majority of mitochondrial acetyl-CoA, and the TCA cycle (via the metabolism of acetyl-CoA) serves as the primary generator of mitochondrial NADH. In 1963, Philip Randle first described a critical balancing act between mitochondrial FAO and glycolysis that prevents mitochondrial NADH overload [60]. Specifically, mitochondrial FAO and glycolysis cannot be simultaneously upregulated [60,61], otherwise mitochondria become overwhelmed by NADH, leading to the toxic shutdown of the tricarboxylic acid (TCA) cycle [62,63,64].

### 1.5. Type 2 Diabetes, Vitamin B6 Insufficiency, and Cancer: Human Studies Strongly Indicate That Flaxseed Is Safe in Populations That Are at Risk of Vitamin B6 Insufficiency

Individuals with type 2 diabetes are already predisposed to risk of vitamin B6 insufficiency, because type 2 diabetes (as a condition) shares a bidirectional, causal relationship with low vitamin B6 bioavailability [65]. Type 2 diabetes promotes low vitamin B6 bioavailability because diabetes accelerates the renal clearance of vitamin B6 [66]. By forcing the attenuation of vitamin B6, diabetes contributes to the worsening of the original diabetic condition. Why? Vitamin B6 performs numerous non-canonical roles that inhibit advanced glycation end-product (AGE) formation [67,68,69], hyperglycemia [70], and oxidative stress [71,72]. However, vitamin B6 cannot perform those biochemical tasks when its bioavailability is low. The result is a worsening of the original diabetic condition, and the vicious cycle between type 2 diabetes and vitamin B6 continues.

A broad sweep across the literature indicates that flaxseed’s anti-vitamin B6 effects are not a threat to at-risk populations (e.g., type 2 diabetics). For example, a meta-analysis of 13 studies on type 2 diabetes suggests that flaxseed is beneficial for HbA1c%, high-density lipoprotein (HDL), and low-density lipoprotein (LDL) [27]. Flaxseed is also beneficial for humans with metabolic syndrome [28,29,30], obesity [24,25], and NAFLD [30,31,32,33]. Does flaxseed hurt individuals who have an exceptionally high risk of vitamin deficiency? A two-year human study of lupus nephritis (a condition where the immune system attacks the nephron and can cause renal failure) suggests that flaxseed consumption decreases serum creatinine and potentially decreases urine microalbumin [73]. Similarly, flaxseed reduces renal injury and attenuates proteinuria in high-risk animal models [74,75]. Flaxseed also exhibits renal benefits in healthy humans [76]. We only found three publications with experimental evidence of increased physiological risk due to flaxseed’s anti-vitamin B6 effects. Those three investigations were designed to test if a vitamin B6 deficient diet predisposes rats to increased risk while consuming flaxseed or 1ADP [48,49,77]. Their conclusion was that a moderate deficiency of vitamin B6 might sensitize humans to the anti-vitamin B6 effects of flaxseed. However, isolated vitamin B6 deficiency is typically rare [78], and flaxseed’s benefits in at-risk human populations are already known (e.g., [24,27,73]).

Regarding cancer, researchers have more recently revealed that type 2 diabetes promotes DNA mutations by enhancing the glycation of nucleotides, histones, and DNA repair proteins, accounting for a portion of diabetic cancer risk [79,80,81,82]. AGEs, which commonly occur in type 2 diabetes, promote carcinogenic transformation by increasing tissue oxidative stress and inducing genome instability [80,83]. Subsequent to carcinogenic transformation, a type 2 diabetic’s elevated blood sugar provides sufficient glucose to satisfy cancer’s common preference for aerobic glycolysis (i.e., Warburg Effect) [84]. Flaxseed’s ability to attenuate diabetic symptoms should help to protect diabetic individuals from these complications. 

### 1.6. Approach and Aim of This Study

We analyze a targeted metabolomics dataset to illustrate the bioenergetic adaptations that occur in White Leghorn laying hens when consuming a flaxseed-supplemented diet. We also conduct a qualitative reanalysis of prior work in [42,85] to help us connect flaxseed’s one-carbon metabolic effects with the bioenergetic adaptations of hens. Our study is not designed to investigate AMPK activation or adenine nucleotide ratios; however, other labs have considered flaxseed as a bioenergetic activator. Researchers illustrated that isolated flaxseed polysaccharide or secoisolariciresinol diglucoside (SDG) can activate AMPK [86,87]. However, neither flaxseed polysaccharide nor SDG are known to antagonize vitamin B6 or to accelerate SAM biosynthesis. Therefore, our model could suggest a novel means by which flaxseed alters bioenergetics, at least in avian species.

## 2. Materials and Methods

### 2.1. Animal Studies and Diet Descriptions

We used single-comb White Leghorn laying hens that were 2.5 years of age. The duration of our diet study was 325 days. All animals were housed in the animal care facility at the University of Illinois in Urbana-Champaign. Hens were grouped in pecks of 5 birds and provided a 17h/7h light/dark cycle. Feed and water were provided ad libitum. Eggs were collected daily from hens and counted by diet group. To assist with a molting phase, a 3 week 12h/12h light/dark cycle was implemented at the beginning of molt. The animal protocol approval assurance number D16-0004 was approved by IACUC on 23 April 2020. The contents and caloric values are shown in Table 1 and Table 2 (published by us in [42]). The following number of hens were assigned to each diet group: control diet (182), 10% defatted flaxseed diet (161), 15% whole flaxseed diet (161), 5% flaxseed oil diet (161), 5% corn oil diet (175), and 5% fish oil diet (165).

### 2.2. Plasma Collection and Animal Necropsy

This protocol was previously published in [42]. We isolated whole blood via wing vein puncture on day 220 of the 325-day study. Blood samples were placed in tubes treated with citrate. Blood was then centrifuged at 2000 rpm for 10 min at 4 °C, and supernatants were transferred to new tubes treated with EDTA. Plasma samples were then stored at −80 °C on the campus of Southern Illinois University, Carbondale. The number of plasma samples that we collected per diet group were as follows: control diet (6), defatted flaxseed diet (5), whole flaxseed diet (6), flaxseed oil diet (6), corn oil diet (6), and fish oil diet (4). One the 325th day of study, we euthanized all animals via CO_2_ asphyxiation and cervical dislocation, and necropsy was performed. Body masses were then recorded. We declared livers as having advanced steatosis if the liver was grossly (>90%) consumed by peanut butter pigmentation [88,89].

### 2.3. LC-MS/MS Analysis of Plasma Metabolites

This protocol is described in detail in [90], and we have described it in [42]. In brief, the Lipid and Metabolite Mass Spectrometry Facility of the University of Texas Southwestern Medical Center analyzed our plasma samples via liquid chromatography tandem mass spectrometry (LC-MS/MS). A targeted metabolite profiling approach was conducted to estimate the relative detection of each metabolite in the sample. The peak area for each metabolite was converted to a variable importance of projection (VIP) score.

### 2.4. Plasma Fatty Acid Methyl Ester (FAME) Gas Chromatography

This protocol is described in [85]. In brief, 2 mL of plasma was isolated from blood that was collected via wing vein puncture and centrifuged. Plasma samples were flash frozen and transferred to Southern Illinois University, Carbondale, and stored at −80 °C. The following number of plasma samples were collected for this analysis: control diet (4), defatted flaxseed diet (4), whole flaxseed diet (4), and flax oil diet (4). Frozen plasma samples were thawed and methylated using a 2-step procedure [91]. A Shimadzu GC-2010 gas chromatograph, equipped with flame ionization detector and a fused silica capillary column was used to measure fatty acid methyl esters (Shimadzu Scientific Instruments Inc., Columbia, MD, USA). Peaks were identified by comparing the retention times versus standards. Heptadecaenoic acid (C17:0) was the internal standard.

### 2.5. Plasma Glucagon Analysis

We utilized a chicken glucagon ELISA kit (MYBiosource #MBS763700) to assay plasma glucagon levels in the plasma of hens that consumed either the control diet or whole flaxseed diet. Seven (7) plasma samples were used from both diets. The ELISA protocol uses a standard sandwich ELISA. A total of 50 µL of plasma template, blank, or standard was added to each well, and 50 µL of biotin-labeled antibody was added (using a 96-well plate). HRP-Streptavidin was then added to each well, and the plate was incubated for 45 min at 37 °C. Plates were then washed and 90 µL of 3,3′,5,5′-Tetramethyl-benzidine (TMB) substrate was added to each well, and the plate was incubated in the dark for 20 min. An amount of 50 µL of stop solution was then added, and spectral transmission was immediately measured at 450 nm. Prior to conducting this assay, we ran several trials to estimate the range of glucagon concentrations in samples, which helped us to calibrate our standard curve. The standard curve for glucagon concentrations (between 0 pg/mL and 50 pg/mL) yielded a coefficient of determination of R^2^ = 0.9971 (Supplementary Figure S8).

### 2.6. Statistical Analysis

Statistical analysis and graphing were performed with R Statistical Software 4.1.3 (R Foundation for Statistical Computing; Vienna, Austria) and R-studio IDE (Rstudio, PBC; Boston, MA, USA). We analyzed plasma metabolites the body masses of non-cancerous hens via one-way ANOVA using Duncan’s multiple range post-test (significant at *p* < 0.05). In hens with advanced liver steatosis, we analyzed body mass via one-way ANOVA using Tukey-Kramer post-test (*p* < 0.05) because of highly variable sample sizes. Prior to conducting one-way ANOVA, we applied the following systematic data transformation to stabilize variance when Bartlett’s statistic was significant (*p* < 0.05). If the median observation was greater than or equal to 1.00, we performed a square root transformation. If the median observation was less than 1.00, we performed an inverse square root transformation (1/sqrt). Odds ratio analysis was conducted to compare advanced liver steatosis in treatment diets versus the control diet (Fisher-exact, *p* < 0.05). A two-tailed *t*-test (assuming unequal variance) was conducted to assess the effect of diet on plasma glucagon (*p* < 0.05). Linear mean regression was used to evaluate the relationship between C3-carnitine and C5-carnitine and between C6-carnitine and C8-carnitine (*p* < 0.05). We removed significant outliers from our statistical tests by using a z-score method of outlier detection (*p* < 0.05). Outliers are noted in the footer of tables, where applicable.

## 3. Results

### 3.1. Effect of Diet on Hen Body Mass

Given the well-known relationship between BMI and AMPK activators (e.g., metformin) [92], we evaluated the effect of flaxseed on hen body mass (Table 3). Our control-fed hens weighed approximately 2.11 kg, while defatted-flaxseed-fed hens and whole-flaxseed-fed hens weighed 1.93 kg and 1.84 kg, respectively (Table 3). Whole-flaxseed-fed hens exhibited significantly lower body mass than defatted-flaxseed-fed hens, indicating a unique effect of whole flaxseed. We previously observed similar results [85].

### 3.2. Pyruvate Metabolism: Evidence for Increased Pyruvate Oxidation via the Pyruvate Dehydrogenase Complex (PDC) in Hens That Consume Defatted Flaxseed

**Figure 2:**
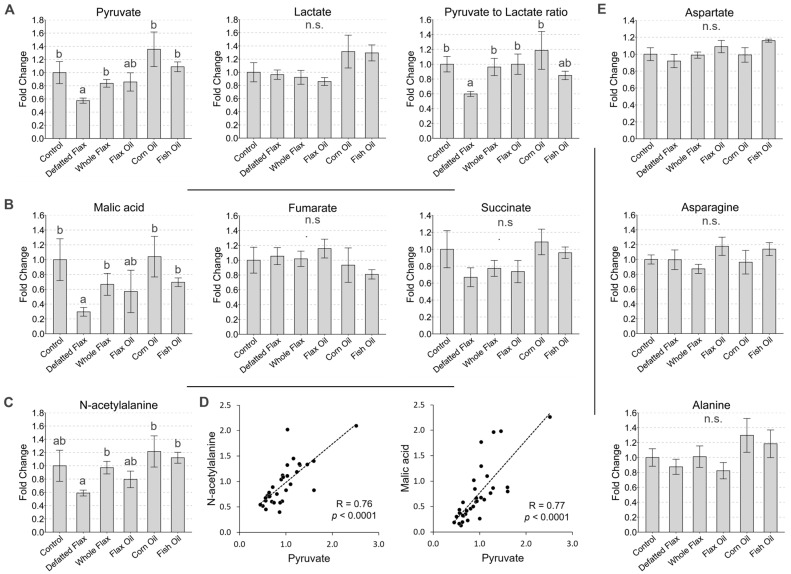
Plasma estimation of pyruvate metabolism. Pyruvate, lactate, and the pyruvate/lactate ratio can be seen in (**A**), in contrast to other metabolites from the TCA cycle (**B**). N-acetylalanine, a product of the spontaneous reaction between pyruvate and ammonia, is displayed (**C**). Scatterplots between pyruvate and N-acetylalanine as well as pyruvate and malic acid are shown (**D**). Lastly, several metabolites that contribute to gluconeogenic production of pyruvate are shown (**E**). LC-MS/MS was used to analyze plasma metabolites. We used one-way ANOVA to analyze VIP scores of metabolites (Duncan’s post-test, *p* < 0.05). We used the following classification system to indicate significant differences: “a” is significant versus “b”; “ab” is not significant versus “a” or “b”; and matching letters are not significantly different (e.g., “ab” versus “ab”). The ANOVA F-test was not significant when “n.s.” is shown. Error bars are SEM. The three black lines in the figure are intended to visually separate subsections.

Pyruvate can be oxidatively decarboxylated via the pyruvate dehydrogenase complex (PDC) to yield acetyl-CoA. Acetyl-CoA then undergoes a rapid condensation with oxaloacetate to yield citrate, via citrate synthase. When PDC is active, oxaloacetate is derived from malate via malate dehydrogenase. When hens consumed defatted flaxseed, plasma pyruvate was attenuated by 43%, concomitantly with a 40% attenuation of the pyruvate/lactate ratio (Figure 2A). Meanwhile, plasma lactate was stable across all diets. Malic acid was attenuated by nearly 70% in hens that consumed defatted flaxseed (Figure 2B). The Pearson correlation between pyruvate and malic acid seemed strong (R = 0.77) (Figure 2D). Succinate and fumarate (two potential TCA cycle intermediates) were unaffected by diet (Figure 2B). N-acetylalanine was 40% lower, on average, in defatted-flaxseed-fed hens (Figure 2C), and the Pearson correlation between pyruvate and N-acetylalanine also seemed strong (R = 0.76) (Figure 2D). The simultaneous attenuation of pyruvate and N-acetylalanine is meaningful because N-acetylalanine is formed through the spontaneous reaction of pyruvate with ammonia. Phenylalanine metabolism can also contribute to the synthesis of N-acetylalanine, but phenylalanine was stable across diets (Supplementary Figure S2). 

Lastly, we asked whether altered gluconeogenesis might be responsible for 43% attenuated pyruvate in hens that consumed defatted flaxseed. The most important gluconeogenic substrate is lactate (via the Cori cycle), in both the liver and kidney of chickens [93]. However, as already mentioned, lactate was stable across diets (Figure 2A). Other gluconeogenic contributors such as aspartate, asparagine, and alanine (Figure 2E), as well as glutamate (Supplementary Figure S2), were stable across diets.

### 3.3. Flaxseed Reduces the Risk of Advanced Liver Steatosis in Laying Hens

**Figure 3:**
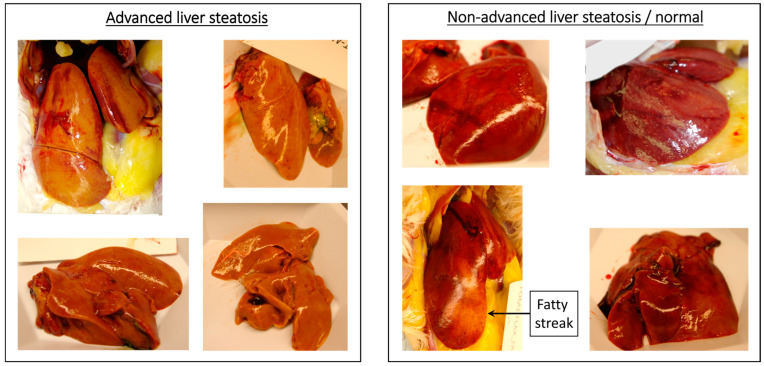
Comparison of laying hen livers with advanced steatosis versus normal appearing liver.

The liver displays an outwardly “brown tan” color (like peanut butter) when it undergoes advanced steatosis [88,89]. In a controlled dietary study, this peanut butter pigmentation can arise, in part, due to jaundicing (i.e., bilirubin accumulation), which is often the result of steatosis-induced liver dysfunction. We classified the hen’s liver as having advanced steatosis when it was consumed (about >90%) by peanut butter pigmentation. See Figure 3 for images that illustrate our classification of advanced steatosis. We detected advanced steatosis in 4.72% of hens consuming whole flaxseed and in 7.76% of hens consuming defatted flaxseed (Table 4). In contrast, 19.84% of hens from the control diet displayed advanced steatosis. Between 10% and 12.75% of hens from the other diets presented advanced steatosis. The odds ratio of advanced steatosis was 0.21 (95% c.i. = 0.07 to 0.52) and 0.34 (95% c.i. = 0.14 to 0.75) in hens consuming whole flaxseed or defatted flaxseed, respectively (Table 4). Notably, in addition, hens consuming corn oil exhibited an odds ratio of 0.45 (95% c.i. = 0.21 to 0.94) for advanced steatosis. Moreover, whole-flaxseed-fed hens with advanced steatosis exhibited an attenuated body mass of 1.65 kg (Table 4), somewhat similar to the mass reduction in Table 3.

### 3.4. Evidence for Upregulated Mitochondrial FAO and Downregulated Lipogenesis in the Livers of Whole-Flaxseed-Fed Hens

We propose that hepatic mitochondrial FAO was upregulated while hepatic lipogenesis was downregulated when hens consumed whole flaxseed. Evidence for that argument includes a 0.21 odds ratio for advanced liver steatosis (Table 4), 60% attenuated plasma aspartate aminotransferase (AST) [94], and 13% attenuated body mass (Table 3). Similarly, in previous work, we observed 14% attenuated body mass, 80% attenuated liver AST, and histological evidence for attenuated liver steatosis when hens consumed whole flaxseed [85]. We discuss additional evidence in Supplemental Text S1 and in Section 4.4.

### 3.5. Acylcarnitines: Evidence for Metabolic Adaptations That Prevent Mitochondrial Overload

**Figure 4:**
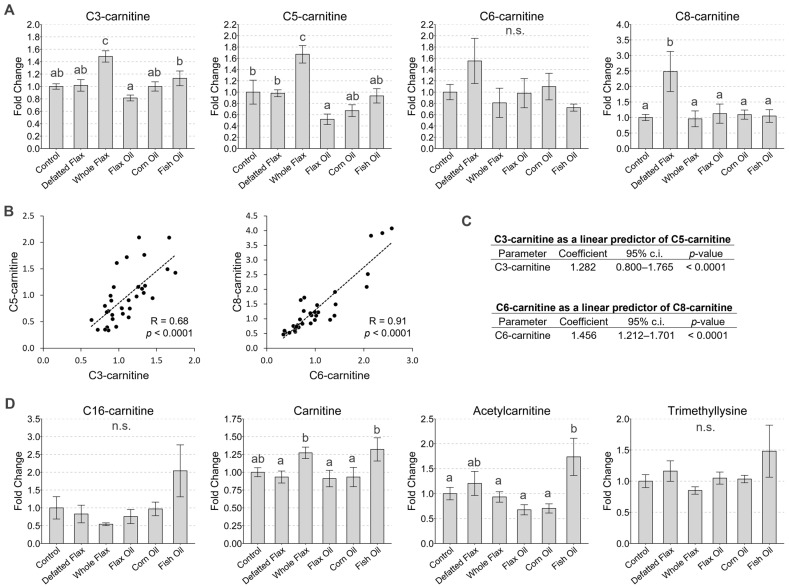
Plasma estimation of acylcarnitine metabolism. C3-, C5-, C6-, and C8-carnitine are shown (**A**) in contrast to the scatterplots between C3- and C5-carnitine, as well as C6- and C8-carnitine (**B**). We display a linear regression output that illustrates C3-carnitine as a predictor of C5-carnitine, and we do the same for C6-carnitine as a predictor of C8-carnitine (**C**). C16-carnitine, carnitine, and molecules that contribute to carnitine synthesis are also shown (**D**). LC-MS/MS was used to analyze plasma metabolites. We used one-way ANOVA to analyze the VIP scores of metabolites (Duncan’s post-test, *p* < 0.05). We used the following classification system to indicate statistically significant differences: “a” is significant versus “b” or “c”; “b” is significant versus “c”; “c” is significant versus “ab”; “ab” is not significant versus “a” or “b”; and matching letters are not significantly different (e.g., “ab” versus “ab”). The ANOVA F-test was not significant when “n.s.” is shown. Error bars are SEM. For C3-carnitine, carnitine, and acetylcarnitine, one outlier was removed from Flax Oil. For C8-carnitine, one outlier was removed from Corn Oil. For C16-carnitine, one outlier was removed from Fish Oil.

Acylcarnitine represents the conjugation of carnitine with a fatty acid of a given carbon length (e.g., C3, C5, etc.). C3- and C5-carnitine were elevated 1.5-fold and 1.7-fold, respectively, in the plasma of hens that consumed whole flaxseed. Meanwhile, we observed a 2.5-fold elevation of plasma C8-carnitine in hens consuming defatted flaxseed, and C6-carnitine was elevated 1.6-fold, on average, in those animals (Figure 4A). Pearson correlations indicated a strong association between C3- and C5-carnitine (R = 0.68) and a very strong association between C6- and C8-carnitine (R = 0.91) (Figure 4B). Interestingly, the 95% confidence interval for the regression coefficient between C6-carnitine and C8-carnitine was greater than one (i.e., 95% c.i. = 1.212 to 1.701), suggesting that the relative rate of C8-carnitine production might exceed the relative rate of C6-carnitine production (Figure 4C). Our colleague, Ralph DeBerardinis, confirmed that C3-, C5-, C6-, and C8-carnitine represent propionate, valerate, hexanoate, and octanoate acylcarnitine, respectively. Lastly, carnitine (i.e., L-Carnitine) was slightly elevated in hens consuming fish oil or whole flaxseed, and acetylcarnitine was elevated when hens consumed fish oil (Figure 4D).

### 3.6. Ornithine Accumulation: Evidence for Flaxseed’s Inhibition of Ornithine Decarboxylase

**Figure 5:**
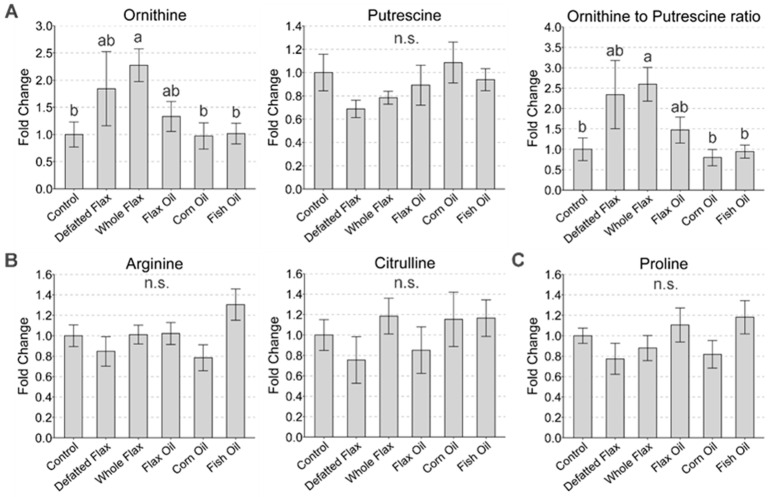
Plasma estimation of ornithine metabolism. Ornithine, putrescine, and the ornithine/putrescine ratio are displayed (**A**) in contrast to arginine and citrulline (**B**) and proline (**C**). LC-MS/MS was used to analyze plasma metabolites. We used one-way ANOVA to analyze the VIP scores of metabolites (Duncan’s post-test, *p* < 0.05). We used the following classification system to indicate statistically significant differences: “a” is significant versus “b”; “ab” is not significant versus “a” or “b”; and matching letters are not significantly different (e.g., “ab” versus “ab”). The ANOVA F-test was not significant when “n.s.” is shown. Error bars are SEM.

Plasma ornithine and the ornithine/putrescine ratio were elevated 2.3-fold and 2.6-fold, respectively, when hens consumed whole flaxseed (Figure 5A). Although not visible in the graph, the two highest plasma ornithine concentrations were recorded in hens consuming defatted flaxseed. Putrescine was, on average, lowest in hens consuming whole flaxseed or defatted flaxseed. Relevant to ornithine’s synthesis in the urea cycle, no effect of diet was observed on arginine or citrulline (Figure 5B) or on fumarate and aspartate (shown above in Figure 2B,E). Lastly, we assessed proline levels because ornithine can be converted to proline via ornithine cyclodeaminase. Diet did not affect proline (Figure 5C).

### 3.7. Flaxseed Induces the Highest HbA1c% Ever Recorded in Birds: The Strangest Paradox

In this sub-chapter, we illustrate that flaxseed induces the highest HbA1c% ever recorded in an avian species. This is a surprising finding given the main thesis of our paper. Unfortunately, we did not measure blood glucose in our hens; however, we suspect that persistent hyperglycemia was not the driver of elevated HbA1c%. We discuss that further in Section 4.9. Blood sugar and blood lipids are regulated uniquely in birds versus mammals [95]. Birds maintain a glycemic state ranging from 200–300 mg of glucose per dL of blood (while fasting) [95,96,97,98]. However, the avian ability to biosynthesize ascorbic acid protects birds from advanced protein glycation and diabetic complication [99,100,101]. 

**Figure 6:**
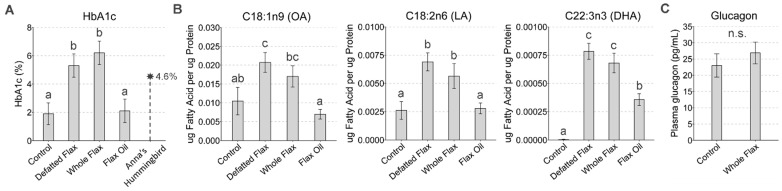
Glycated hemoglobin, plasma free fatty acids, and plasma glucagon in laying hens. The glycated hemoglobin (HbA1c%) values of our hens were previously analyzed in [85] (**A**). The HbA1c% of Anna’s Hummingbird was published in [98] and is shown here for comparison purposes (**A**). Plasma fatty acid methyl esters (*n* = 4 per group) were measured via gas chromatography (these samples were derived from the same hens in (**A**), but this is their first publication) (**B**). Plasma glucagon was analyzed in hens via ELISA (*n* = 7 per group) (**C**). One-way ANOVA was used to compare group differences (Duncan’s post-test, *p* < 0.05) (**B**). In (**C**), Student’s *t*-test was used to compare differences (*p* < 0.05). We used the following classification system to indicate statistically significant differences: “a” is significant versus “b” or “c”; “b” is significant versus “c”; “bc” is significant versus “a”; “ab” is not significant versus “a” or “b”; “bc” is not significant versus “ab”; and matching letters are not significantly different (e.g., “a” versus “a”). The statistical test was not significant when “n.s.” is shown. Error bars are SEM.

In 1998, the Anna’s Hummingbird was documented with an HbA1c% of 4.6%, representing the highest HbA1c% recorded in birds at that time (Figure 6A, adapted from [98]). Then, in 2016, our lab observed that defatted flaxseed and whole flaxseed, respectively, cause White Leghorn laying hens to display an HbA1c% of 5.3% and 6.3% (Figure 6A, [85]). Those HbA1c% values are the highest HbA1c% ever documented in birds. In that same study, our control-fed hens displayed an HbA1c% of 1.9% (Figure 6A, [85]). Simultaneously, oleic acid (OA), linoleic acid (LA), and docosahexaenoic acid (DHA), were elevated in the blood of our flaxseed-fed laying hens (Figure 6B, [85]). These observations motivated a hypothesis that flaxseed increases glucagon secretion into blood, because glucagon activates hepatic glycogenolysis and renal gluconeogenesis, thereby inducing hyperglycemia in chickens [95,102,103]. Surprisingly, our ELISA results indicated no effect of flaxseed on plasma glucagon (Figure 6C). We then considered the possible role of ascorbic acid. Plasma ascorbic acid was attenuated by 50% in our hens that consumed defatted flaxseed versus control-fed hens (Supplementary Figure S2). Notably, plasma ascorbic acid was attenuated by 78% in hens that consumed defatted flaxseed versus whole-flaxseed-fed hens. Therefore, the attenuation of ascorbic acid might account, at least partially, for elevated HbA1c% when hens consume defatted flaxseed. Insulin was not evaluated because chickens are considered insulin insensitive [95,104,105,106].

## 4. Discussion and Conclusions

### 4.1. Does Our Work Address Liver Metabolism, and Is Our Work Reproducible in Humans?

The liver of a White Leghorn hen represents about 3.5% to 4% of total body mass [107], whereas in humans, the liver represents 1.5% to 2% of body mass [108,109]. The highly representative mass of liver in White Leghorn should qualify our plasma study as a valid proxy for liver metabolism. Can we reproduce the findings of our current study in humans? Recently, we received encouraging information from our collaborator, Susan McCann, who informed us that the one-carbon metabolic framework that we developed in [42] helps to shed light on the plasma metabolomics of post-menopausal women who consume dietary flaxseed (private communication).

### 4.2. Defatted Flaxseed’s Effect on Glucose Uptake, Glycolysis, and PDC Activity in the Liver: Have We Discovered “Avian Metformin”?

Why was plasma pyruvate attenuated by 43% when hens consumed defatted flaxseed, while whole flaxseed exerted no effect? This is easier to understand through the Randle cycle, where the oscillation between FAO and glycolysis is regulated by the availability of fatty acids [61]. The high-fat content of whole flaxseed would promote mitochondrial FAO, whereas glycolysis would be favored by the low-fat content of defatted flaxseed. 

**Figure 7:**
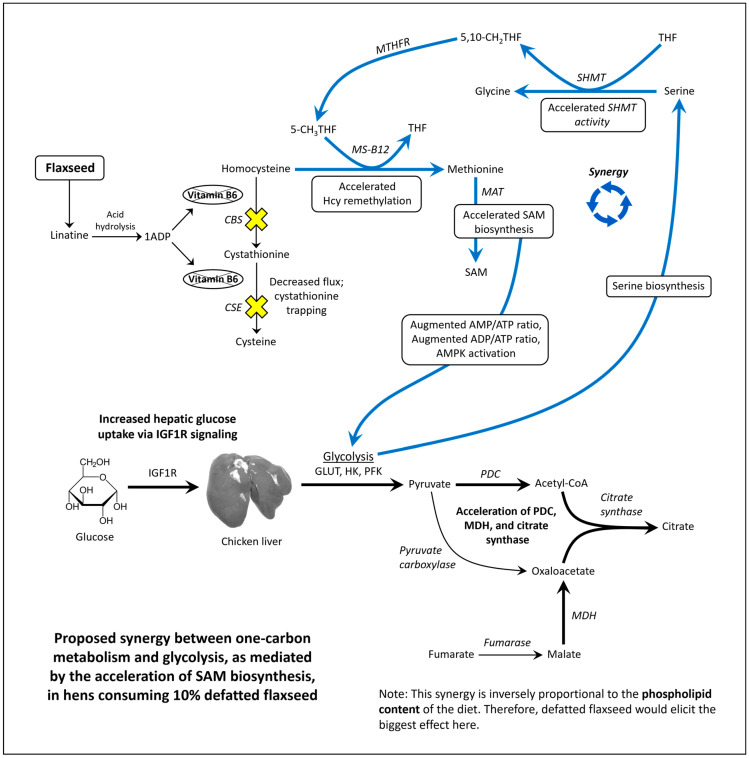
Model of the synergy between one-carbon metabolism and glycolysis in hens consuming 10% defatted flaxseed. 1ADP = 1-amino D-proline, 5-CH_3_THF = 5-methyl tetrahydrofolate, 5,10-CH_2_THF = 5,10-methylene tetrahydrofolate, AMPK = AMP-activated protein kinase, CBS = cystathionine beta synthase, CSE = cystathionase, GLUT = glucose transporter, HK = hexokinase, Hcy = homocysteine, IGF1R = insulin-like growth factor receptor 1, MAT = methionine adenosyltransferase, MDH = malate dehydrogenase, PDC = pyruvate dehydrogenase complex, MTHFR = methlyene tetrahydrofolate reductase, PFK = phosphofructokinase, SAM = S-adenosylmethionine, SHMT = serine hydroxymethyltransferase, THF = tetrahydrofolate.

Our data suggest that defatted flaxseed causes PDC acceleration in hens. The most important criteria were: 43% attenuated pyruvate, 40% attenuated pyruvate/lactate ratio, and 70% attenuated malic acid. When hens consumed defatted flaxseed, the pyruvate/lactate was attenuated in the presence of stable lactate. The stability of lactate suggests that an insult to glycolytic flux did not cause pyruvate’s attenuation. Defatted flaxseed’s ability to cause a 70% attenuation of malic acid is very meaningful here. In the TCA cycle, malate is converted to oxaloacetate via malate dehydrogenase (MDH), followed by the condensation of oxaloacetate with acetyl-CoA through citrate synthase (Figure 7). Citrate synthase (by condensing oxaloacetate with acetyl-CoA) regulates the clearance of acetyl-CoA from the mitochondrial matrix. The clearance of acetyl-CoA is critical because acetyl-CoA inhibits PDC. Indeed, researchers identified a multi-enzyme complex consisting of MDH bound to citrate synthase, whereby oxaloacetate is spontaneously transferred to the catalytic subunit of citrate synthase, which enables the rapid condensation of oxaloacetate with acetyl-CoA [110]. The presence of this “MDH—citrate synthase” complex suggests that PDC acceleration occurs at the major expense of malate (because malate is the primary source of oxaloacetate) and pyruvate. Therefore, it is very logical to observe 70% attenuated malic acid with 43% attenuated pyruvate. Malic acid’s attenuation was unlikely due to fumarase deceleration because fumarate accumulation did not occur. Overall, we hypothesize that 10% defatted flaxseed enhances hepatic TCA cycle flux by accelerating the activities of PDC, MDH, and citrate synthase in laying hens.

Hepatic PDC acceleration should prompt an increase of hepatic glucose uptake. In chickens, glucose uptake is regulated almost entirely by insulin-like growth factor 1 (IGF1) signaling [95,104,105,106]. Our lab’s defatted-flaxseed-fed hens expressed 3.25-fold and 2.05-fold upregulated hepatic mRNA transcripts for IGF1 and IGF1-binding protein (IGF1BP), respectively [85]. A chicken’s plasma IGF1 protein level is regulated by hepatic IGF1 mRNA transcription [111,112]; therefore, our defatted-flaxseed-fed hens should have increased plasma IGF1 protein levels. This would enable increased glucose uptake in the liver. Further supporting increased glucose uptake, our lab’s defatted-flaxseed-fed hens displayed 3.61-fold downregulated hepatic mRNA transcripts for protein tyrosine phosphatase 1 (PTP1) [85]. PTP1 is a phosphatase that inhibits the IGF1 receptor (IGF1R) [113]. Downregulated PTP1 might indicate increased potential for IGF1R signal transduction. These observations provide good evidence that hepatic glucose uptake should be upregulated via IGF1 signaling when hens consume 10% defatted flaxseed.

Can gluconeogenesis explain the 43% attenuation of pyruvate in defatted-flaxseed-fed hens? We argue no, mainly because lactate was not affected by diet. Lactate is the primary gluconeogenic substrate in chicken liver and kidney under fed and fasted conditions [93]. Additionally, pyruvate is a poor substrate for gluconeogenesis in chicken liver because chicken hepatocytes lack functional cytosolic phosphoenolpyruvate carboxykinase (PEPCK-c) [93,103]. Pyruvate is gluconeogenic mostly in the renal tubule of the chicken; however, lactate is still two- to threefold more gluconeogenic than pyruvate in the renal tubule [93]. We did not see an effect of diet on glycine [42], serine [42], glutamate (Supplementary Figure S2), alanine, asparagine, or aspartate. Those amino acids contribute slightly to gluconeogenesis in the renal tubule of the chicken [93]. We also did not observe an effect of flaxseed on plasma glucagon, which is important given glucagon’s impact on gluconeogenesis [102,103]. Overall, we conclude that increased gluconeogenesis is very unlikely to account for defatted flaxseed’s 43% attenuation of plasma pyruvate.

Future research will be designed to investigate glucose uptake, glycolytic flux, and PDC activity, in response to defatted flaxseed supplementation or 1ADP monotherapy in laying hens. Such investigation could reveal 1ADP’s utility as a homologue for “avian metformin”.

### 4.3. Synergy between One-Carbon Metabolism and Glycolysis When Hens Consume 10% Defatted Flaxseed: Implications for Aging and Longevity in Avian Species

Our current study suggests a link between glycolysis and one-carbon metabolism when hens consume defatted flaxseed. In our prior work [42], we illustrated that defatted flaxseed should increase metabolic flux through the folate cycle, according to 37% attenuated dimethylglycine (DMG), 30% attenuated serine (on average), and 40% attenuated serine/glycine ratio (on average). Importantly, the attenuated serine/glycine ratio was due to an attenuation of serine coupled with an increase of glycine, which imitates the catabolism of serine via serine hydroxymethyltransferase (SHMT) (Figure 7). Increased consumption of serine via SHMT would compromise serine bioavailability, and thereby sensitize the serine biosynthesis pathway toward activation. The serine biosynthetic pathway branches from glycolysis when 3-phosphoglycerate is metabolized through a three-step process that yields serine [114,115]. Moreover, only two enzymes separate 3-phosphoglycerate from pyruvate, indicating that serine biosynthesis and PDC are very proximal. The oscillation between PDC and serine biosynthesis would occur at a minimal metabolic cost.

A major limiting factor for flux through PDC and serine biosynthesis is glucose availability (downstream of glucose 6-phosphate). We have evidence that hepatic flux through the glucose 6-phosphate pathway (i.e., glycolysis) could be increased in hens that consume defatted flaxseed. These animals exhibited 85% attenuated plasma glucuronate [42], concomitantly with 55% attenuated plasma ascorbic acid (Supplementary Figure S2). Glucuronic acid (i.e., protonated glucuronate) and ascorbic acid are synthesized through the glucose-1-phosphate pathway. Therefore, defatted flaxseed might divert carbon flux away from the glucose 1-phosphate pathway as a means to enhance glycolytic flux toward PDC and serine biosynthesis. Our work with defatted flaxseed could offer an important contribution to the study of aging and longevity. Researchers observed that the attenuation of plasma glucuronic acid functions as a biomarker for increased health-span and lifespan in humans and mice [116]. Similarly, we observed attenuated glucuronate and increased longevity in defatted-flaxseed-fed hens [42]. We propose that the attenuation of plasma glucuronic acid (as a biomarker for decreased aging) indicates a preferential flux of glucose toward serine biosynthesis and PDC. Others have also proposed serine’s critical role in the regulation of aging and health-span [117,118].

Our hypothesis is that defatted flaxseed’s anti-vitamin B6 effects activate energy sensors (via accelerated SAM biosynthesis) in a manner that synergizes glycolysis with one-carbon metabolism (Figure 7). This synergy would promote increased glucose flux toward serine biosynthesis and PDC, and thereby maintain sufficient levels of serine and ATP.

### 4.4. We Propose That Flaxseed Attenuates Body Mass and Hepatic Steatosis by Upregulating Hepatic Mitochondrial FAO and Downregulating Hepatic Lipogenesis in Hens

Chickens, like mammals, leverage liver-specific AMPK to prevent liver steatosis and liver disease [119,120]. In previous work, our whole-flaxseed-fed hens displayed 2.88-fold and 2.10-fold downregulated liver mRNA transcripts for sterol regulatory binding factor (SREBF1) and fatty acid synthase (FASN) [85]. SREBF1 is a transcription factor that regulates the transcription of sterol biosynthetic genes, while FASN (which is transcribed by SREBF1) physically executes de novo fatty acid synthesis. Other researchers observed similar molecular effects in association with hepatic AMPK gene expression in laying hen livers [119]. 

The 13% decreased body mass, 0.21 odds ratio for advanced liver steatosis, and 60% attenuation of plasma AST, suggest that hepatic mitochondrial FAO is accelerated and hepatic lipogenesis is decelerated in whole-flaxseed-fed hens. We produced nearly identical results in a previous study [85]. One limitation of our odds ratio analysis for advanced liver steatosis is that we only conducted necropsy on animals that survived the entire 325-day study. See [42] for hen survival information. We do not suspect that a mortality bias influenced our odds-ratio analysis for advanced liver steatosis, with the exception of the corn-oil-fed hens (they exhibited increased mortality).

**Figure 8:**
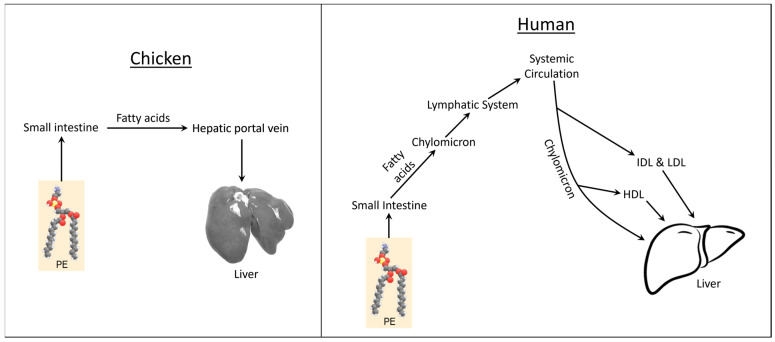
Comparison of fatty acid transport from the small intestine to the liver (chicken versus human). HDL = high density lipoprotein, IDL = intermediate density lipoprotein, LDL = low density lipoprotein, PE = phosphatidylethanolamine.

Chickens are uniquely vulnerable to developing fatty liver because a chicken’s hepatic portal vein transports dietary fatty acids directly to the liver [121] (Figure 8). As such, chicken liver gets “first crack” at metabolizing dietary fatty acids. Additionally, de novo lipogenesis is restricted almost entirely to the liver in chickens, which further increases fatty liver risk [122]. In theory, it would be nearly impossible for a chicken’s extrahepatic tissues to maintain static lipid volume when hepatic mitochondrial FAO is accelerated and hepatic lipogenesis is decelerated. Supporting this, researchers showed that dietary flaxseed causes a dose-dependent and time-dependent attenuation of abdominal adiposity in chickens and ducks [123,124,125,126]. Humans are protected from fatty liver because chylomicrons and lipoprotein molecules transport dietary fatty acids through lymph and systemic circulation before delivering them to the liver [127,128] (Figure 8). Additionally, lipogenesis is distributed more evenly between extrahepatic tissues and the liver in humans.

### 4.5. Synergy between One-Carbon Metabolism and Phosphatidylcholine Catabolism in Hens Consuming a 15% Whole Flaxseed Supplemented Diet

We did not assess feed intake in our study; therefore, someone might ask if body mass was reduced by decreased feed intake. Numerous studies indicate that 10% flaxseed and 15% flaxseed have no effect on feed intake in White Leghorn laying hens [129,130,131,132]. Further supporting stable feed intake, our whole-flaxseed-fed hens and defatted-flaxseed-fed hens yielded the first and third highest daily egg laying rate amongst the six diets, respectively (Supplementary Figure S3). We propose that flaxseed’s augmentation of one-carbon metabolism gave rise to the body mass attenuation. When hens consumed whole flaxseed, plasma choline was attenuated between 39% to 52% (95% c.i), suggesting a tremendous increase in choline catabolism [42]. According to our model from [42], we argue that whole flaxseed causes the hyperactivation of betaine homocysteine methyltransferase (BHMT), as a compensation for severely perturbed transsulfuration. However, BHMT hyperactivation increases the catabolism of betaine, and betaine must be synthesized from choline (or acquired from increased dietary consumption). The majority of choline is stored in phosphatidylcholine in plasma membrane. To increase the bioavailability of soluble choline, the cell leverages phospholipases such as phospholipase D1 (PLD1) [133]). Phospholipases, during the process of liberating choline from phosphatidylcholine, also liberate non-esterified free fatty acids (NEFAs) in the cytosol. Intracellular NEFAs must, in large part, undergo mitochondrial FAO to prevent lipotoxic effects. Therefore, BHMT hyperactivation should increase PLD1 activity and mitochondrial FAO, culminating in the attenuation of hepatic lipid mass. The activation of mitochondrial FAO would be complimented by the inhibition of lipogenesis, thereby further attenuating hepatic lipid mass.

**Figure 9:**
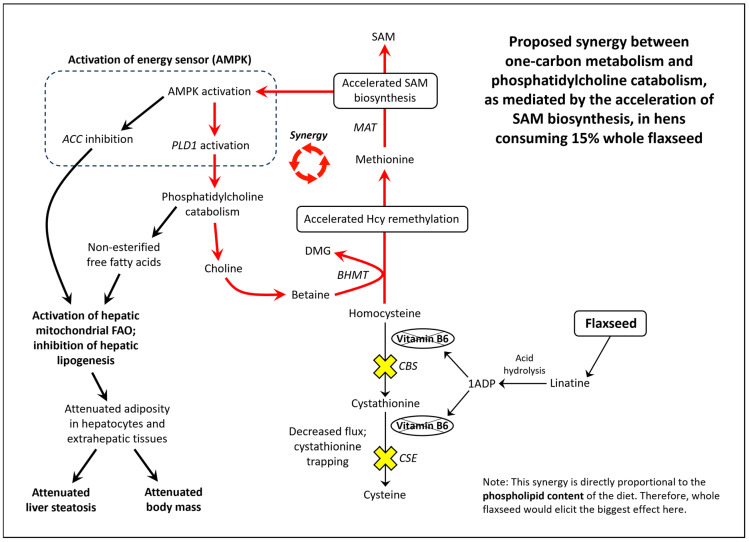
Model of the synergy between one-carbon metabolism and phosphatidylcholine catabolism in hens consuming 15% whole flaxseed. 1ADP = 1-amino D-proline, ACC = acyl-CoA carboxylase, AMPK = AMP-activated protein kinase, BHMT = betaine homocysteine methyltransferase, CBS = cystathionine beta synthase, CSE = cystathionase, DMG = dimethylglycine, FAO = fatty acid oxidation, Hcy = homocysteine, MAT = methonine adenosyltransferase, PLD1 = phospholipase D1, SAM = S-adenosylmethionine.

Researchers have already shown that SAM acceleration activates AMPK [50]. AMPK activation, in turn, promotes PLD1 activation and ACC inhibition, resulting in increased NEFA bioavailability and mitochondrial FAO [55,56,133]. Through this perspective, our one-carbon metabolic framework in [42] synergizes with our current work to suggest the presence of a “BHMT/MAT/AMPK/PLD1” cycle when hens consume 15% whole flaxseed (Figure 9).

### 4.6. Acylcarnitine Synthesis: Protecting Mitochondria from Metabolic Overload When Bioenergetic Pathways Are Accelerated

We observed 2.5-fold elevated C8-carnitine coincident with 43% attenuated pyruvate in hens consuming defatted flaxseed. We propose that elevated C8-carnitine is a direct marker for increased peroxisomal FAO, resulting from the attenuation of mitochondrial FAO. Peroxisomes partially oxidize long-chain fatty acids to a medium-chain length [134,135], providing an appropriate substrate for the synthesis of medium-chain acylcarnitines (e.g., C8-carnitine). A major stimulator of peroxisomal FAO is the cell’s ability to detect mitochondrial overload [136,137]. In the presence of accelerated PDC activity, the mitochondria would detect elevated acetyl-CoA and elevated NADH, thereby shifting the oxidation of long-chain fatty acids to peroxisomes. The peroxisomal oxidation of long-chain fatty acids is an important contributor to cytosolic malonyl-CoA [138,139,140], and malonyl-CoA (as a CPT1 inhibitor) blocks the mitochondrial oxidation of long-chain fatty acids [141]. The synthesis of medium-chain acylcarnitines increases in peroxisomes when long-chain FAO shifts from the mitochondria to the peroxisomes [137,142]. We propose that the routing of FAO to the peroxisomes was the mechanism behind elevated C8-carnitine. The balancing act between mitochondrial FAO and peroxisomal FAO illustrates a key element of the Randle cycle, whereby mitochondrial FAO must be balanced with glycolysis [143]. Mitochondrial FAO, if unrestricted during PDC acceleration, would overwhelm mitochondria with NADH and cause TCA cycle shutdown [62,63,64].

We observed elevated C3- and C5-carnitine in the plasma of hens consuming whole flaxseed. C3- and C5-carnitine are represented by propionic acid and valeric acid, both of which are branch chain amino acids (BCAAs). Propionic acid and valeric acid support anaplerotic flux into the TCA cycle via propionyl-CoA and valeryl-CoA [144]. Cells prevent the anaplerotic flux of propionic acid and valeric acid through the conversion of propionyl-CoA and valeryl-CoA to C3-carnitine and C5-carnitine, respectively. In other words, the formation of C3- and C5-carnitine diverts BCAAs away from the TCA cycle and attenuates mitochondrial NADH synthesis [62,63,64]. Our hypothesis is that whole-flaxseed-fed hens leverage BCAA-carnitine formation to prevent mitochondrial NADH overload in the presence of accelerated mitochondrial FAO. Inversely to this idea, researchers illustrated that the attenuation of mitochondrial FAO accelerates the anaplerotic flux of BCAAs into the TCA cycle [145].

### 4.7. Evidence for Whole Flaxseed’s Inhibition of Ornithine Decarboxylase (ODC): A Potential Biomarker for Highly Elevated AMPK Activity

Experimental evidence suggests that AMPK activators inhibit ornithine decarboxylase (ODC), thereby causing the accumulation of ornithine [57,58]. ODC is the rate limiting enzyme for polyamine biosynthesis, via the conversion of ornithine to putrescine. The polyamine biosynthetic pathway is bioenergetically expensive because polyamines are essential for cell growth [146,147,148]. Therefore, AMPK conserves ATP by inhibiting ODC. We observed 2.3-fold elevated plasma ornithine and a 2.6-fold elevated ornithine/putrescine ratio in hens consuming whole flaxseed. This supports a hypothesis that whole flaxseed inhibits ODC by activating AMPK. We evaluated the levels of 5-Aminoimidazole-4-carboxamide ribonucleoside (AICAR) across diets, because AICAR is a potent activator of AMPK. There was no effect of diet on AICAR (Supplementary Figure S2). Surrogate indicators of urea cycling (i.e., arginine, citrulline, fumarate, succinate) and renal output (reported in [42]) seem unlikely to explain elevated ornithine levels in whole-flaxseed-fed hens. Someone might argue that ODC activity was attenuated via 1ADP’s anti-vitamin B6 effects because PLP serves as ODC’s cofactor. We do not support that argument because defatted-flaxseed-fed hens displayed the biggest vitamin B6 perturbation [42], yet ornithine was not significantly elevated in those animals. Instead, we propose that whole flaxseed strongly activates hepatic AMPK to yield attenuated ODC activity. Moreover, our current findings refute a hypothesis that we made in [42], where we stated that defatted flaxseed possibly upregulates polyamine biosynthesis. We made that hypothesis because defatted-flaxseed-fed hens displayed elevated plasma methylthioadenosine (MTA) [42], and MTA is a byproduct of polyamine biosynthesis. However, we were unaware that MTA is a potent inhibitor of spermidine synthase and spermine synthase [149,150]. Therefore, we currently hypothesize that defatted flaxseed attenuates polyamine biosynthesis, in part, via elevated MTA in hens.

### 4.8. The Exaggerated Catabolic Phenotype of Whole-Flaxseed-Fed Hens Might Be Explained by the Phosphatidylethanolamine Methyltransferase (PEMT) Pathway

Whole flaxseed might leverage the phosphatidylethanolamine methyltransferase (PEMT) pathway to enhance the AMP/ATP ratio and ADP/ATP ratio, thereby promoting the high activation of energy sensors (e.g., AMPK). We illustrate this phenomenon in Supplementary Text S2 and Supplementary Figures S4 and S5 [29,42,51,52,54,151,152,153,154,155,156,157,158,159,160,161,162,163,164].

### 4.9. Flaxseed and Elevated HbA1c%: Possibly the Result of 1ADP’s Anti-Vitamin B6 Effect

Clinicians commonly measure a patient’s hemoglobin A1c percentage (HbA1c%) to estimate whether the individual was exposed to persistent hyperglycemia during the previous 120 days [165,166]. Humans are considered potentially pre-diabetic when HbA1c% exceeds 5.7%, and type 2 diabetes is suspected when HbA1c% exceeds 6.5% [167]. Humans display a normal blood glucose of 100 mg/dL, whereas laying hens display a higher, normal blood glucose of 300 mg/dL [168]. Despite having high glucose, chickens have a low HbA1c% ranging from less than 1.0% to slightly less than 2.0% [85,169].

Our flaxseed-fed hens in [85] displayed the highest HbA1c% levels ever observed in birds. Our finding of increased HbA1c% is puzzling because flaxseed attenuates HbA1c% in humans [27]. Under normal conditions, chicken glycemia is regulated by glycogenolysis and gluconeogenesis, with glucagon acting as the hormonal regulator [95]. Flaxseed exerted no effect on plasma glucagon, suggesting that glycogenolysis and gluconeogenesis should be stable. Additionally, all gluconeogenic metabolites (besides pyruvate) were stable across diets. Unfortunately, we did not measure blood glucose in our animals, so we cannot be certain if hyperglycemia was involved. However, the literature provides good evidence that flaxseed does not induce hyperglycemia in laying hens. Poultry researchers illustrated that blood glucose is stable when adult Hy-Line laying hens consume diets supplemented with either 9%, 18%, or 27% flaxseed, versus a control diet [169]. If anything, their results suggest a decreasing linear trend in blood glucose as the percentage of flaxseed increases [169]. Additional evidence for normal glycemia in our flaxseed-fed hens was improved liver function, reduced hepatic steatosis risk, and reduced NAFLD risk (in our current work and in [85,94]). Hyperglycemia associates directly with liver dysfunction in humans [170,171], and so our liver results suggest normal glycemia in flaxseed-fed hens.

As we discussed in our introduction, vitamin B6 helps to prevent protein glycation [67,68,69]. Therefore, 1ADP might be a major culprit behind elevated HbA1c% in flaxseed-fed hens. Several unique components might be important here. The White Leghorns’ liver is large in comparison to its total body mass, about two-fold larger in comparison to humans [107,108,109]. This implies that the hen’s red blood cells experience increased exposure to the liver environment, per volume of blood. Additionally, the high metabolic rate of avian species should accelerate spontaneous chemical reactions (e.g., glycation reactions), which might account for why evolution favored ascorbic acid biosynthesis in avian species [101]. We, therefore, hypothesize that proteins undergo increased glycation in the livers of flaxseed-fed White Leghorn hens, due to the effect of vitamin B6 antagonism in combination with large liver size and high metabolic rate. This idea could potentially account for the elevated HbA1c% of flaxseed-fed hens. Lastly, the attenuation of plasma ascorbic acid might also associate with elevated HbA1c% [100]. Future studies will be conducted to determine if supplementation with pyridoxine or vitamin C can attenuate HbA1c% in flaxseed-fed laying hens.

### 4.10. Proposed Therapeutic Model in Humans

**Figure 10:**
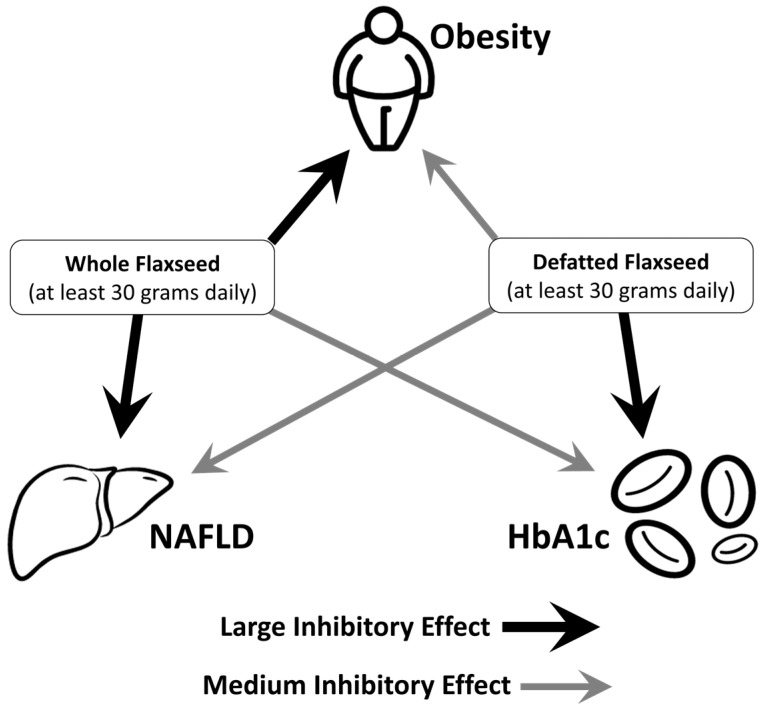
Model of flaxseed’s therapeutic effects on obesity, NAFLD, and HbA1c.

In Figure 10, we indicate that whole flaxseed’s biggest effect should be the attenuation of obesity and NAFLD, whereas defatted flaxseed should be most effective at attenuating type 2 diabetic complications. Supplementary Figures S6 and S7 illustrate the bioenergetic mechanisms behind these outcomes.

## Figures and Tables

**Table 1:** Diets and associated ingredients (g/100 g of diet).

Ingredient (g/100 g)	Control	10% Defatted Flaxseed Meal	15% Whole Flaxseed	5% Flax Oil	5% Corn Oil	5% Menhaden Fish Oil
Corn	67.40	54.90	47.58	52.00	52.00	52.00
Soybean Meal	18.30	18.30	18.30	18.30	18.30	18.30
Whole Flaxseed			15.00			
Corn Gluten Meal	3.00			5.00	5.00	5.00
Corn Oil					5.00	
Flax Oil				5.00		
Fish Oil						5.00
Defatted Flaxseed Meal		10.00				
Qual Fat		3.80	2.50			
Solka Floc	0.30	2.00	5.62	8.70	8.70	8.70
Each diet received the following in g/100 g of diet: Limestone (8.75), Dical (1.5), Salt (0.3), Vitamin Mix ^1^ (0.2), Mineral Mix ^2^ (0.15) and DL-Methionine (0.1)						

^1^ Vitamin premix (per kg of diet): retinyl acetate, 4400 IU; cholecalciferol, 25 mg; DL-a-tocopheryl acetate, 11 IU; vitamin B12, 0.01 mg; riboflavin, 4.41 mg; D-Capantothenate, 10 mg; niacin, 22 mg; and menadione sodium bisulfite, 2.33 mg. ^2^ Mineral premix (mg/kg of diet): Mn, 75 from MnO; Fe, 75 from FeSO_4_·7H_2_O; Zn, 75 from ZnO; Cu, 5 from CuSO_4_·5H_2_O; I, 0.75 from ethylene diamine dihydroiodide; and Se, 0.1 from Na_2_SeO_3_.

**Table 2:** Calculations of nutrient percentages and total energy of diets.

Calculated Analysis	Control	10% Defatted Flaxseed Meal	15% Whole Flaxseed	5% Flax Oil	5% Corn Oil	5% Menhaden Fish Oil
TME ^1^, kcal/kg	2816	2816	2815	2815	2815	2815
CP ^2^, % TME	16.56	17.04	16.50	16.49	16.49	16.49
Calcium, % TME	3.73	3.77	3.75	3.73	3.73	3.73
aPhosphorus ^3^, % TME	0.38	0.40	0.38	0.37	0.37	0.37
Met + Cys, % TME	0.67	0.72	0.64	0.67	0.67	0.67

^1^ TME= total metabolizable energy, ^2^ CP= crude protein, ^3^ aPhosphorous= available phosphorous.

**Table 3:** Body mass of non-cancerous hens, by diet (adapted from [42]).

Parameter	Control	Defatted Flax	Whole Flax	Flax Oil	Corn Oil	Fish Oil
Body Mass (kg)	2.11	1.93	1.84	2.10	2.07	2.02
Significance Group	c	b	a	c	c	bc
Standard Deviation	0.33	0.29	0.28	0.31	0.35	0.32
Sample Size (n)	86	85	71	84	62	70

Notes: One-way ANOVA was used to evaluate differences (Duncan’s post-test, *p* < 0.05). Significant differences are indicated when groups lack a similar letter (e.g., “b” versus “bc” is not significantly different). One outlier was removed from the whole flax diet.

**Table 4:** Risk of advanced liver steatosis by diet.

Diet Group	Hens with Advanced Liver Steatosis (*n* Hens)	Total Hens at Necropsy (*n* Hens)	Percentage of Hens with Advanced Liver Steatosis	Odds Ratio of Advanced Liver Steatosis, versus the Control Diet (Odds Ratio, 95% c.i.)	Mean Body Mass (kg) of Hens with Advanced Liver Steatosis (mean ± sd)
Control	25	126	19.84%	NA	2.16 ± 0.36 (b)
Defatted Flax	9	116	7.76%	0.34 (0.14, 0.75)	2.04 ± 0.26 (ab)
Whole Flax	5	106	4.72%	0.21 (0.07, 0.52)	1.65 ± 0.26 (a)
Flax Oil	13	102	12.75%	0.59 (0.28, 1.21)	2.13 ± 0.34 (ab)
Corn Oil	12	120	10.00%	0.45 (0.21, 0.94)	1.91 ± 0.28 (ab)
Fish Oil	11	103	10.68%	0.49 (0.22, 1.03)	2.25 ± 0.42 (b)

Notes: Odds ratio estimates were significant (*p* < 0.05) when the 95% confidence interval did not overlap with 1.00. One-way ANOVA was used to evaluate differences in body mass (Tukey–Kramer post-test, *p* < 0.05). We used the following classification system to indicate statistically significant differences: “a” is significant versus “b”; “ab” is not significant versus “a” or “b”; and matching letters are not significantly different (e.g., “ab” versus “ab”).

## Data Availability

All data presented in this article are available on request from the corresponding author. The data are not publicly available due to privacy.

## Supplementary Materials

The following supporting information can be downloaded at: https://www.mdpi.com/article/10.3390/metabo13080945/s1. Figure S1. Model illustrating how the MAT reaction potentiates AMP binding to the regulatory subunits of energy-sensing proteins in the cytosol. Figure S2. Plasma estimation of ascorbic acid, AICAIR, glutamate, inosine, and phenylalanine. Figure S3. Effect of diet on egg laying performance. Figure S4. Model of the phosphatidylethanolamine methyltransferase (PEMT) pathway as a means to additively enhance the AMP/ATP ratio and ADP/ATP ratio, in liver of whole-flaxseed-fed hens. Figure S5. Estrogen response element (ERE) and AP-1 enhancers within the regulatory region of *Gallus gallus* PEMT. Figure S6. Model of 10% defatted flaxseed’s major metabolic effects in avian liver, assuming hepatic AMPK activation. Figure S7. Model of 15% whole flaxseed’s major metabolic effects in avian liver, assuming hepatic AMPK activation. Figure S8. Plasma glucagon standard curve. Text S1. Additional evidence for accelerated mitochondrial FAO in the livers of whole-flaxseed-fed hens. Text S2. The phosphatidylethanolamine methyltransferase (PEMT) pathway might explain the exaggerated catabolic phenotype of whole-flaxseed-fed hens.

## Abbreviations

1ADP	1-amino D-proline
ACC	Acetyl-CoA carboxylase
ADK	Adenosine kinase
ADP	Adenosine diphosphate
AICAR	5-aminoimidazole-4-carboxamide-1-β-D-ribofuranoside
AMP	Adenosine monophosphate
AMPK	AMP-activated protein kinase
AST	Aspartate aminotransferase
ATP	Adenosine triphosphate
BCAA	Branch-chain amino acid
C3, C5, C6, C8, C16-carnitine	Acylcarnitines with either 3, 5, 6, 8, or 16 carbons in the fatty acid
CBS	Cystathionine beta synthase
CSE	Cystathionase
dAMP	Deoxyadenosine monophosphate
DHA	Docosahexaenoic acid
DNA	Deoxyribonucleic acid
ERE	Estrogen response element
FAO	Fatty acid oxidation (mitochondrial)
FASN	Fatty acid synthase
HbA1c%	Glycated hemoglobin (percentage)
HDL	High density lipoprotein
IDL	Intermediate-density lipoprotein
IGF1	Insulin-like growth factor 1
IGF1BP	Insulin-like growth factor 1 binding protein
IGF1R	Insulin-like growth factor 1 receptor
LA	Linoleic acid
LC-MS/MS	Liquid chromatography tandem mass spectrometry
LDL	Low-density lipoprotein
MAT	Methionine adenosyltransferase
MDH	Malate dehydrogenase
mRNA	Messenger RNA
MS-B12	Methionine synthase complexed with vitamin B12
NADH	Nicotine adenine dinucleotide (protonated form)
NAFLD	Non-alcoholic fatty liver disease
NCBI	National Center for Biotechnology Information
OA	Oleic acid
ODC	Ornithine decarboxylase
PC	Phosphatidylcholine
PDC	Pyruvate dehydrogenase complex
PE	Phosphatidylethanolamine
PEMT	Phosphatidylethanolamine methyltransferase
PTP1	Protein tyrosine phosphatase 1
SAH	S-adenosylhomocysteine
SAHH	S-adenosylhomocysteine hydrolase
SAM	S-adenosylmethionine
SREBF1	Sterol regulatory binding factor 1 (aka SREBP1)
VIP	Variable importance of projection score
