# Supplementary material for: Flaxseed Reduces Cancer Risk by Altering Bioenergetic Pathways in Liver: Connecting SAM Biosynthesis to Cellular Energy
Source: Metabolites. 2023 Aug 14;13(8):945. doi: 10.3390/metabo13080945 (PMC10456508; doi:10.3390/metabo13080945)
Supplement: Supplementary file 1 [file metabolites-13-00945-s001.zip › metabolites-2501797-supplementary material.pdf]

metabolites

Supplementary materials

Flaxseed Reduces Cancer Risk by Altering Bioenergetic Pathways in Liver: Connecting SAM Biosynthesis to Cellular Energy

William C. Weston [1], Karen H. Hales [2] and Dale B. Hales [1,2,*]

Table of Contents

Supplementary Texts

Text S1. Additional evidence for accelerated mitochondrial FAO in the liver's of whole-flaxseed-fed hens.

Text S2. The phosphatidylethanolamine methyltransferase (PEMT) pathway might explain the exaggerated catabolic phenotype of whole-flaxseed-fed hens.

Supplementary Figures

Figure S1. Model illustrating how the MAT reaction promotes increased AMP binding to the regulatory subunits of energy-sensing proteins in the cytosol.

Figure S2. Plasma estimation of ascorbic acid, AICAIR, glutamate, inosine, and phenylalanine.

Figure S3. Effect of diet on egg laying performance.

Figure S4. Model of the phosphatidylethanolamine methyltransferase (PEMT) pathway as a means to additively enhance the AMP/ATP ratio and ADP/ATP ratio in livers of whole-flaxseed-fed hens.

Figure S5. Estrogen response element (ERE) and AP-1 enhancers within the regulatory region of Gallus gallus PEMT, according to NCBI Genbank.

Figure S6. Model of 10% defatted flaxseed's major metabolic effects in avian liver, assuming hepatic AMPK activation.

Figure S7. Model of 15% whole flaxseed's major metabolic effects in avian liver, assuming hepatic AMPK activation.

Figure S8. Plasma glucagon standard curve.

Supplementary Texts

Text S1. Additional evidence for accelerated mitochondrial FAO in the liver's of whole-flaxseed-fed hens.

Recall that avian species biosynthesize ascorbic acid. The on-average elevated ascorbic acid in whole-flaxseed-fed hens might indicate increased oxygen demand (Supplementary Figure S2). Ascorbic acid is essential for gut iron absorption, and iron is required for hemoglobin formation. Hemoglobin, in turn, delivers oxygen to mitochondria, which supports electron flux through the electron transport chain (ETC). Mitochondrial FAO is a major source of NADH, and NADH-derived electrons reduce oxygen at complex 4 of the ETC. Ascorbic acid biosynthesis, by supporting hemoglobin formation, is directly tied to ETC activity. Interestingly, researchers showed that iron supplementation increases ascorbic acid biosynthesis in chickens [151]. Ascorbic acid also helps to mitigate reactive oxygen species, which would be important when mitochondrial respiration is increased.

Inversely, the 50% attenuated plasma ascorbic acid of defatted-flaxseed-fed hens (versus the control diet) might indicate decelerated mitochondrial FAO. This idea aligns with our model of accelerated flux through the pyruvate dehydrogenase complex (PDC) in defatted-flaxseed-fed hens. Notably, ascorbic acid was attenuated 78% in defatted-flaxseed-fed hens versus whole-flaxseed-fed hens.

Other metabolites could be relevant here. Our whole-flaxseed-fed hens in [42] displayed slightly attenuated plasma taurine. Taurine is catabolized during mitochondrial FAO as a means to maintain redox homeostasis and acyl dehydrogenase activity [152,153]. The slightly increased plasma carnitine in whole-flaxseed-fed hens (in Figure 4) might also indicate increased CPT1 activity [54].

Text S2. The phosphatidylethanolamine methyltransferase (PEMT) pathway might explain the exagerrated catabolic phenotype of whole-flaxseed-fed hens

The phosphatidylethanolamine methyltransferase (PEMT) pathway could additively enhance the AMP/ATP ratio and ADP/ATP ratio in whole-flaxseed-fed hens (Supplementary Figure S4). PEMT hyperactivation (in conjunction with accelerated SAM biosynthesis) should promote the very high activation of energy sensing proteins like AMPK. This could account for the profoundly catabolic phenotypes of our whole-flaxseed-fed hens. Almost all PEMT activity takes place in liver, which makes our discussion about PEMT appropriate [154]. The only prerequisite for PEMT hyperactivation is the simultaneous elevation of phosphatidylethanolamine (PE) and SAM [155]. Whole flaxseed is a rich source of PE, and our previous work indicates that flaxseed accelerates SAM biosynthesis [42]. By elevating the bioavailability of SAM and PE, the whole flaxseed diet is optimal for accelerating PEMT activity. Additionally, the chicken's ability to transport fatty acids directly through the hepatic portal vein would increase PE formation in liver (Figure 8).

The activities of PEMT and S-adenosylhomocysteine hydrolase (SAHH) are causally associated [155-157], because PEMT is the largest known generator of S-adenosylhomocysteine (SAH). In turn, SAH must be hydrolyzed by SAHH, which yields adenosine and homocysteine. PEMT hyperactivation and SAHH hyperactivation go hand in hand, because SAHH activity is required to prevent SAH's inhibition of PEMT. We previously observed numerous biomarkers of SAHH hyperactivation in whole-flaxseed-fed hens [42]. Specifically, we observed 4-fold elevated adenosine, 6-fold elevated adenosine/SAH ratio, and an elevated homocysteine/SAH ratio when hens consume whole flaxseed. We also observed slightly elevated deoxyadenosine monophosphate (dAMP) [42], possibly indicating increased adenosine kinase (ADK) activity. Increased dAMP is important here because adenosine can be monophosphorylated by ADK to yield AMP. Increased AMP production would increase the AMP/ATP ratio. Additionally, the ADK reaction consumes ATP to yield ADP, which increases the ADP/ATP ratio. In essence, the hyperactivation of PEMT should increase the AMP/ATP ratio and the ADP/ATP ratio, suggesting PEMT's potent role as a bioenergetic enzyme.

Adenosine can also be metabolized to inosine via adenosine deaminase; however, plasma inosine was stable across diets (Supplementary Figure S2). As such, adenosine was more likely metabolized via ADK (according to elevated dAMP in [42]) in whole-flaxseed-fed hens.

Can PEMT hyperactivation be achieved in humans? As already mentioned, PEMT hyperactivation requires increased bioavailability of SAM and PE. If flaxseed's anti-vitamin B6 effects accelerate SAM biosynthesis in humans, the decisive factor would be hepatic PE availability. Hepatic PE availability can be increased, in part, by increasing the concentration of high-density lipoprotein (HDL) in blood. Importantly, HDL molecules are enriched with PE [158]. In humans, the blood HDL concentration can be augmented via dietary flaxseed (30 grams daily) [27], niacin monotherapy [159], and yoga exercise [160]. The fact that flaxseed increases blood HDL concentrations is extremely meaningful. Statins should also increase hepatic PE availability because statins increase the concentration of PE within plasma membranes [161]. Estrogen therapy might also provide a novel means to increase hepatic PEMT activity [162] because the DNA regulatory region of human PEMT contains multiple incomplete estrogen response elements (EREs) [163,164]. Even more pronounced, the DNA regulatory region of chicken PEMT contains a complete ERE palindrome in the first intron (Supplementary Figure S5).

Supplementary Figures

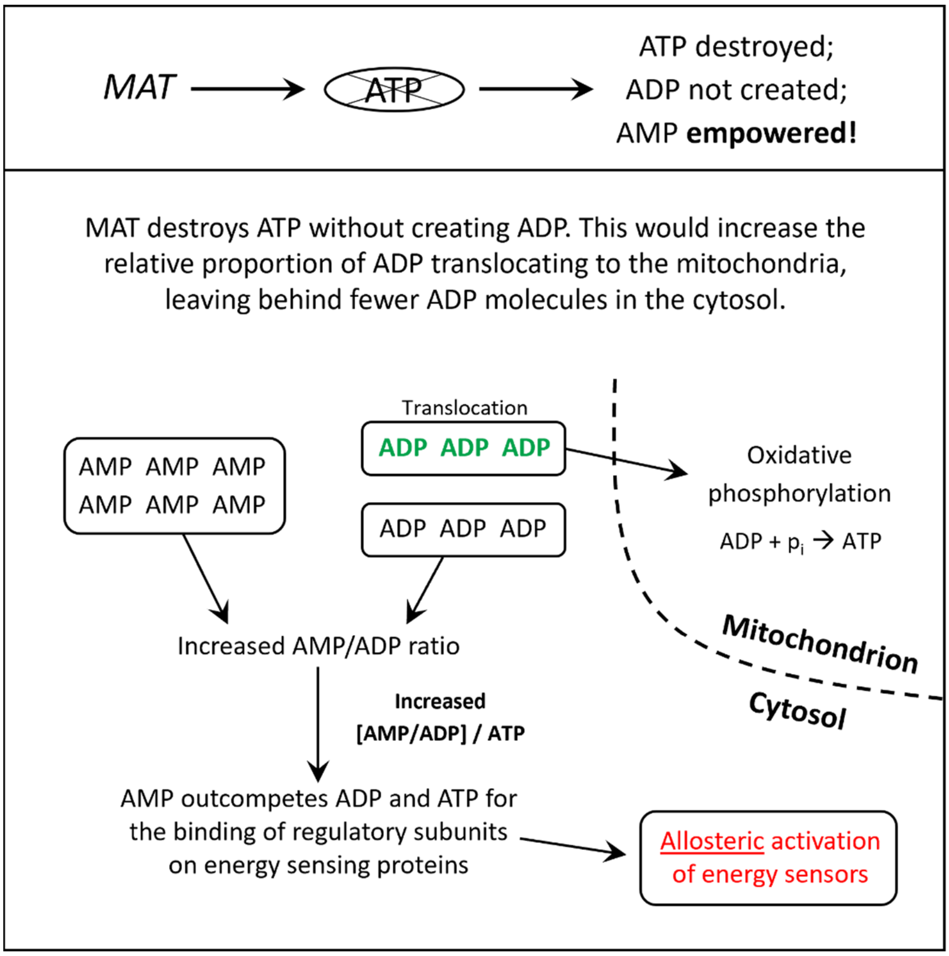

Figure S1. Model illustrating how the MAT reaction promotes increased AMP binding to the regulatory subunits of energy-sensing proteins in the cytosol. In turn, this would increase the allosteric activation of energy sensors like AMPK [51,52].

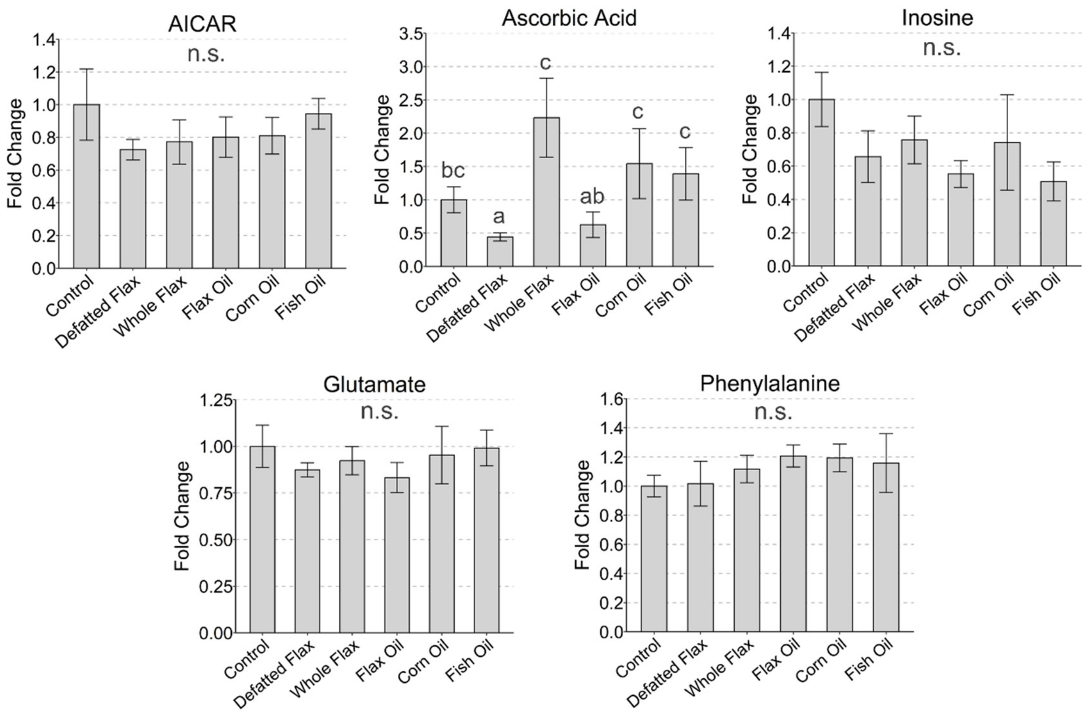

Figure S2. Plasma estimation of AICAIR, ascorbic acid, glutamate, inosine, and phenylalanine. LC-MS/MS was used to analyze plasma metabolites. We used one-way ANOVA to analyze VIP scores of metabolites (Duncan's post-test, $p<0.05$). Significant differences are shown as follows: "a" is significantly different from "bc" and "c"; "ab" is significantly different from "c"; and all other comparisons are not significantly different. The ANOVA F-test was not significant when "n.s." is shown. Error bars are SEM. For ascorbic acid, one outlier was removed from Control.

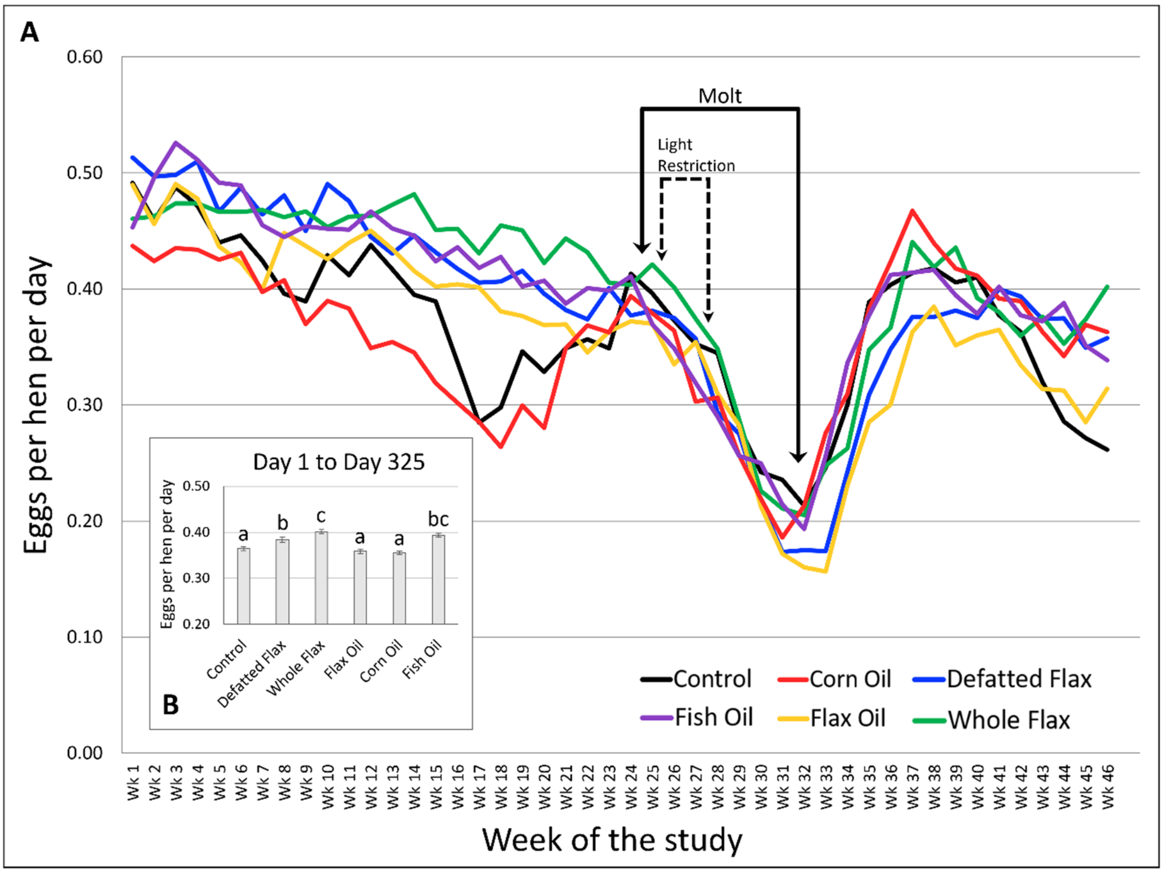

Figure S3. Effect of diet on egg laying performance. In this figure, we illustrate the weekly average of daily eggs laid per hen by diet group (**A**). Also shown in the graph is the cumulative effect of diet on daily egg laying during the entire 325-day study (**B**). In (**A**), we used one-way ANOVA and Tukey-Kramer post test ($p < 0.05$). Eggs were collected from each diet group daily, and the average daily egg laying per hen was calculated as follows: the total number of eggs collected per diet group (that day) divided by the total number of living hens within the diet group (that day). In (**B**), "a" is significantly different from "b", "c", or "bc". "b" is significantly different from "c". "bc" is not significantly different from "c" or "b".

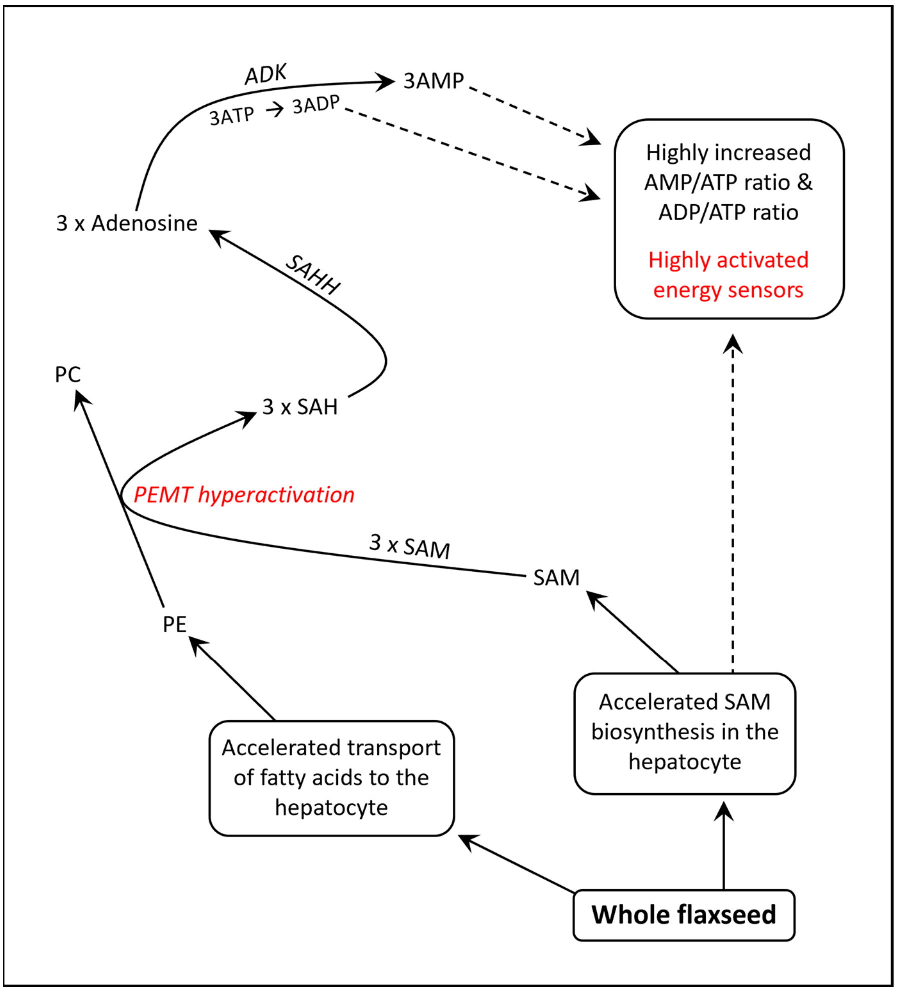

Figure S4. Model of the phosphatidylethanolamine methyltransferase (PEMT) pathway as a means to additively enhance the AMP/ATP ratio and ADP/ATP ratio, in liver of whole-flaxseed-fed hens. ADK = adenosine kinase, PC = phosphati-dylcholine, PE = phosphatidylethanolamine, SAH = S-adenosylhomocysteine, SAHH = S-adenosylhomocysteine hydrolase, SAM = S-adensylmethionine. Dashed lines infer an augmentation of the AMP/ATP ratio and ADP/ATP ratio.

NCBI, Genbank
-Version & Accession: AADN05000376
-*PEMT* (*Gallus gallus*), NP_001006164.1, GRCg6a, Chromosome 14, 5190747:5228149

The estrogen response element (ERE) is found from 5′ 5227643 to 5227655 3′ and begins 424bp downstream from the transcription start site, in intron 1.

ERE: 5′ (5227643) **CCAGTACCACTGG** (5227655) 3′

AP-1 enhancers (TGA G/C TCA) were found in the *PEMT* locus both upstream (-) and downstream (+) of the transcription start site.

1: TGAGTCA (-5,530bp)
2: TGACTCA (-1,510bp)
3: TGACTCA (+29,211bp)
4: TGAGTCA (+33,674bp)
5: TGAGTCA (+42,485bp)

Figure S5. Estrogen response element (ERE) and AP-1 enhancers within the regulatory region of Gallus gallus PEMT, according to NCBI Genbank.

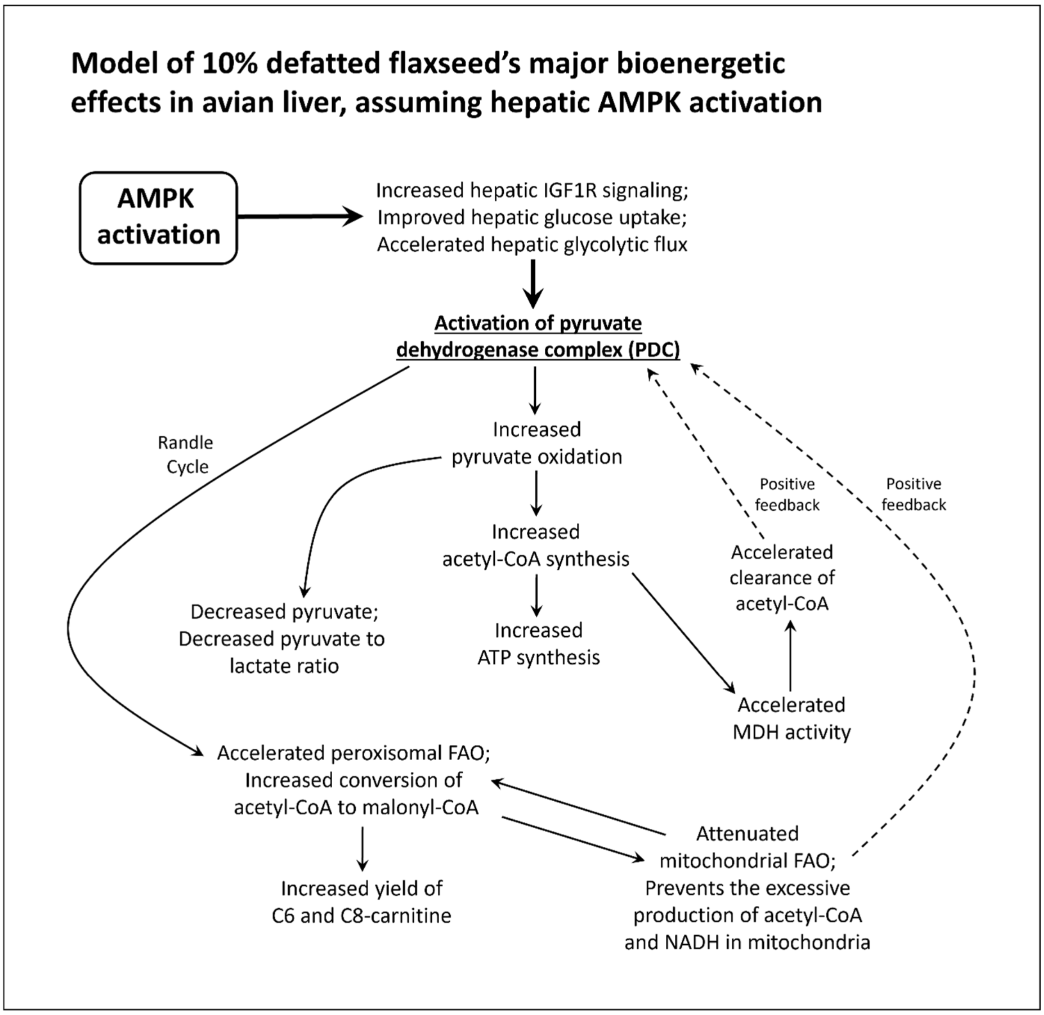

Figure S6. Model of 10% defatted flaxseed's major bioenergetic effects in avian liver, assuming hepatic AMPK activation.

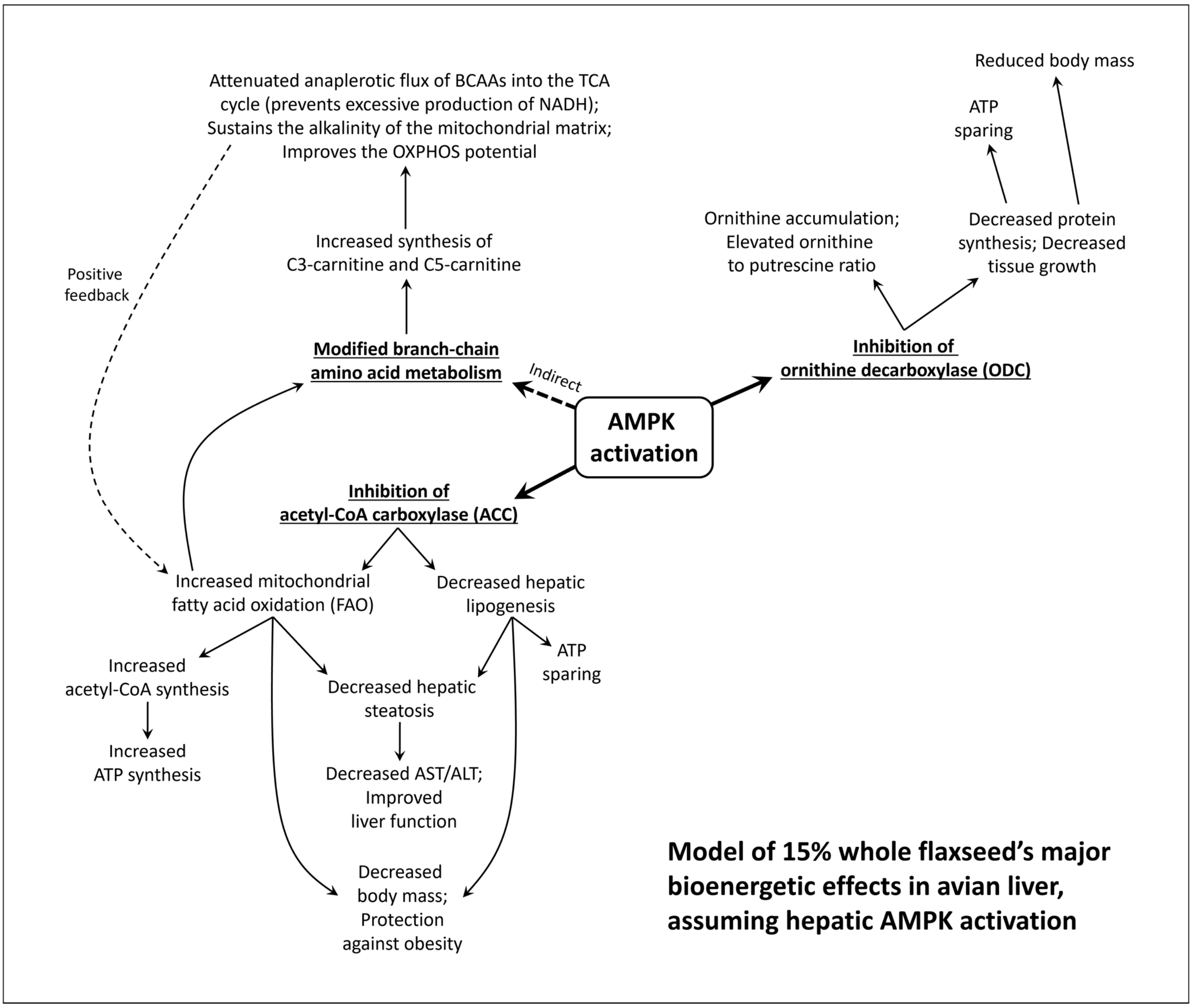

Figure S7. Model of 15% whole flaxseed's major bioenergetic effects in avian liver, assuming hepatic AMPK activation.

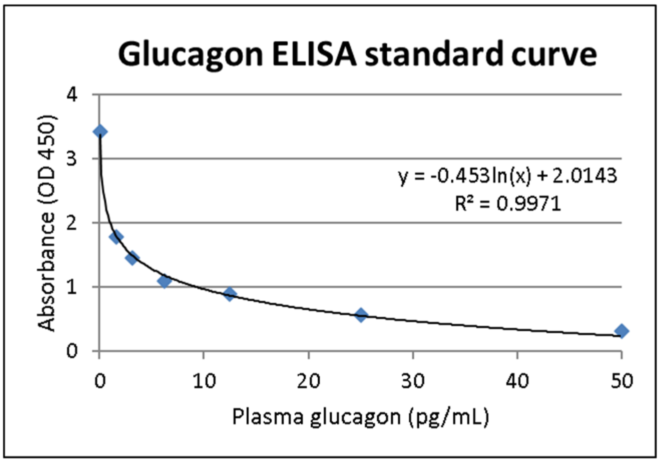

Figure S8. Plasma glucagon standard curve.
